# Photoelectrochemical performance of a spin coated TiO_2_ protected BiVO_4_-Cu_2_O thin film tandem cell for unassisted solar water splitting†

**DOI:** 10.1039/d2ra05774c

**Published:** 2022-11-02

**Authors:** S. R. Sitaaraman, Andrews Nirmala Grace, Raja Sellappan

**Affiliations:** School of Electronics Engineering, Vellore Institute of Technology Vellore 632014 India; Centre for Nanotechnology Research, Vellore Institute of Technology Vellore 632014 India raja.sellappan@vit.ac.in

## Abstract

A tandem cell consisting of a Mo-BiVO_4_/TiO_2_/FeOOH photoanode–Cu_2_O/TiO_2_/MoS_2_ photocathode was prepared for unassisted solar water splitting. The protective TiO_2_ layer was prepared by a cost-effective spin coating technique. The individual Mo-BiVO_4_/TiO_2_/FeOOH photoanode and the Cu_2_O/TiO_2_/MoS_2_ photocathode yielded a current density of ∼0.81 mA cm^−2^ at 1.23 V *vs.* RHE and ∼−1.88 mA cm^−2^ at 0 V *vs.* RHE, respectively under 100 mW cm^−2^ xenon lamp illumination. From the individual photoelectrochemical analysis, we identify the operating points of the tandem cell as 0.66 V *vs.* RHE and 0.124 mA cm^−2^. The positive current density from the operating points proves the possibility of non-zero operation of the tandem cell. Finally, a two-electrode Mo-BiVO_4_/TiO_2_/FeOOH-Cu_2_O/TiO_2_/MoS_2_ tandem cell was constructed and analysed for unassisted operation. The obtained unassisted current density of the tandem cell was ∼65.3 μA cm^−2^ with better stability compared to the bare BiVO_4_-Cu_2_O tandem cell. The results prove that the spin coated TiO_2_ protective layer can be a viable approach to protect the photoelectrodes from photocorrosion with better stability and enhanced photoelectrochemical (PEC) performance.

## Abstract 2

Mo-BiVO_4_/TiO_2_/FeOOH photoanode–Cu_2_O/TiO_2_/MoS_2_ photocathode tandem cells with photoelectrochemical stability testing.

## Graphical abstract

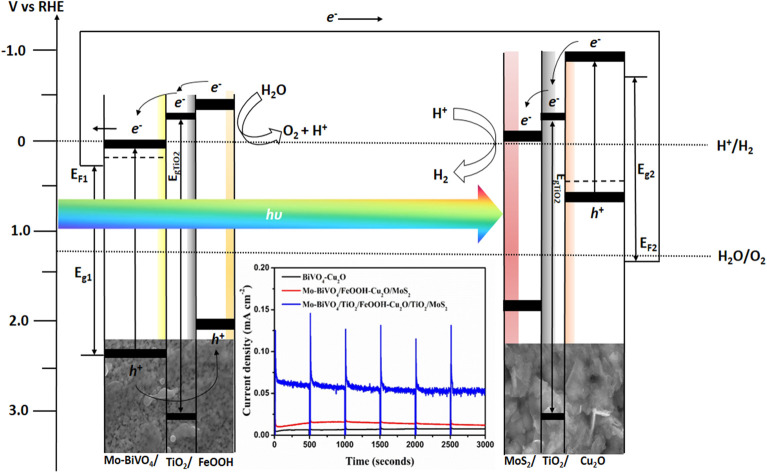

## 1. Introduction

Solar hydrogen is considered as a promising form of green energy for the future due to its potential to mitigate global warming and provide sustainable energy throughout the day despite the intermittency of sunlight.^1^ The search for an efficient practical semiconductor photocatalyst material has been pursued since the pioneering work done by Fujishima and Honda in 1972.^2^ The identification of a suitable single photoelectrode system with good chemical stability and long-term durability is still quite challenging without external bias. The combination of mature solar cell technology and electrolyser^3^ can be an alternative approach to meet the practical requirements but the system complexity and the cost per kilogram of hydrogen produced will be high.^4^ Direct conversion of solar energy using a tandem photoelectrochemical cell (PEC) is a cost-effective approach for unassisted solar water splitting.^5^ In the tandem or z-scheme approach,^6^ complementary semiconductors are chosen in which the photoanode or top electrode (where oxidation takes place) should be a wide bandgap semiconductor and the photocathode or the bottom electrode (where reduction takes place) should be a narrow bandgap semiconductor.^7,8^ The light that is transmitted by the photoanode will be absorbed by the photocathode so that the optical absorption can be maximized to the entire incoming solar spectrum. Since two photoelectrodes are used with straddled band edges, there would be a sufficient amount of photopotential developed for the splitting of water unlike in single electrode systems.^9,10^ The photoelectrodes can be chosen according to the modified contour plot devised by M. S. Prévot *et al.*, in order to achieve a maximum theoretical efficiency.^11^

In the literature, several combinations of semiconductor electrode materials have been used in tandem cell. Out of different materials, BiVO_4_ (ref. 12) has often been used as the photoanode because of its availability, favorable bandgap and the stable improved photocurrent.^13^ P. Xu *et al.*, constructed BiVO_4_ photoanode with Si nanoarray photocathode. The photoanode was enhanced by Mo doping and adding co-catalyst Co-Pi. Similarly, the photocathode was p-Si grown in the form of nanoarray loaded with Pt *via* photo electrodeposition. The tandem cell produced ∼0.46 mA cm^−2^ photocurrent at zero bias condition in linear sweep voltammetry.^52^ However, the stability tests of the cell was challenging because of the p-Si nanoarray degradation in aqueous solution.^14^ CIGS and CZTS are similar in structure and have a variable bandgap of 1 eV to 1.7 eV.^15,16^ The narrow bandgap CIGS and CZTS are excellent choices with BiVO_4_ in PEC tandem cell. M. Chen *et al.*, used BiVO_4_/NiOOH/FeOOH as the photoanode and CIGS/CdS/Al_2_O_3_/TiO_2_/Pt as the photocathode and they achieved current density of 0.82 mA cm^−2^ for 30 minutes in 1 M potassium borate solution at pH 9.2.^17^ Although the performance of CIGS tandem cell showed some promise, but the use of toxic materials is an issue in case of CIGS. A tandem cell consisted of BiVO_4_ photoanode with CZTS/CdS/HfO_2_/Pt photocathode was prepared which produced a stable current density of ∼0.68 mA for over 10 hours.^18^ In CIGS^19^ and CZTS,^20^ a CdS interfacial layer should be deposited to improve the transfer of charges between the electrode and electrolyte interfaces. The deposition of number of layers with ultrathin thickness is also difficult in terms of large area testing. Another choice of p-type material has been CuBi_2_O_4_ which has a bandgap of about 1.3 to 1.8 eV and has a positive flat band potential of 1 V *vs.* RHE.^21^ There are several reports on BiVO_4_ photoanode and CuBi_2_O_4_ photocathode tandem structure proposed in the literature with considerable improvement in the PEC performance.^22–24^

Another choice of photocathode has been Cu_2_O^25^ which has narrow bandgap of 2.0 eV with a band edges suitable for reduction. P. Bornoz *et al.*,^27^ constructed the first ever BiVO_4_-Cu_2_O tandem cell. In this work, photoelectrochemical properties with respect to the optical property of the photoelectrode was analyzed. The photocathode consisted of Cu_2_O/Al:ZnO/TiO_2_/RuO_*x*_ while the photoanode chosen was BiVO_4_ with variable thickness in order to enhance the absorbance of the photocathode. The best performance was obtained for 200 nm thick BiVO_4_ photoanode and the 500 nm thick^26^ modified Cu_2_O photocathode. The tandem cell produced current density of ∼0.1 mA cm^−2^ for 4000 seconds with the STH efficiency of 0.4%.^27^ Similarly L. Pan *et al.*, enhanced the performance of Cu_2_O photocathode and constructed tandem PEC cell with BiVO_4_ photoanode. Cu_2_O was protected by n-type Ga_2_O_3_ overlayers for efficient charge separation and protection. The photocathode Cu_2_O/Ga_2_O_3_/TiO_2_/NiMo was combined with hydrogen treated Mo-BiVO_4_/NiFeO_*x*_ co-catalyst. The tandem cell produced current density of 2.4 mA cm^−2^ and the highest solar to hydrogen efficiency of ∼3% was achieved for the tandem cell combinations.^28^ From the above studies, it was observed that serious modifications in BiVO_4_ and Cu_2_O are required to obtain better performance in unassisted tandem cell operation. Particularly, the TiO_2_ protective layer is important for a stable PEC operation. The ALD process^29^ used in several studies is quite complex and a time-consuming process which should be replaced by a low cost process with better quality. X. Yin *et al.*, analyzed the nanostructured BiVO_4_/TiO_2_/FeOOH electrodeposited Cu_2_O tandem cell performance. The stability of unassisted tandem cell was assessed and the current density of 0.06 mA cm^−2^ was achieved. The study revealed poor stability of materials as a result of Cu_2_O photocorrosion^30^ since no interfacial layers are employed to enhance the PEC performance.^31^ X. Fu *et al.*, made Cu_2_O photocathode loaded with hydrogen treated Ti_3_C_2_T_*x*_ MXene and tested the performance in tandem with BiVO_4_ photoanode. Cu_2_O was grown on Cu foam and MXene was loaded on the Cu foam in order to improve the oxygen vacancies on the photocathode. The increased oxygen vacancies improve the charge transport to the surface and the conductivity. The tandem cell was illuminated from the Cu_2_O photocathode side contrary to the conventional illumination from the anode side. The STH efficiency of 0.55% was obtained for this tandem cell configuration.^32^ Deposition of protective layer and loading co-catalysts are effective strategies for unassisted tandem PEC cell. This dual approach can reduce the overpotential of the semiconductors used in the tandem cell and therefore the STH efficiency can be improved.

In this work, we have chosen semiconductor oxide materials BiVO_4_ as a photoanode and Cu_2_O as a photocathode and this combination can produce a maximum efficiency of close to 9% according to the contour plot.^11^ Since both oxide materials have been individually characterized and well-documented in the literature, it is appropriate to construct a tandem cell with the aim to examine the effect of spin-coated TiO_2_ protective layer on the PEC performance. Molybdenum was doped in BiVO_4_ to increase the conductivity while FeOOH and MoS_2_ was loaded on the photoanode and photocathode as co-catalysts, respectively. The individual performance of BiVO_4_ photoanode and Cu_2_O photocathode was analyzed and the linear sweep voltammetry response was overlaid to find the operating points of the tandem PEC cell. Finally, Mo-BiVO_4_/TiO_2_/FeOOH-Cu_2_O/TiO_2_/MoS_2_ tandem structure was tested for unassisted tandem cell operation and a stable photocurrent response was obtained. No work has been hardly explored on spin coated TiO_2_ protective layer for both photoanode and photocathode. We believe that the results would contribute to the further advancement in the unassisted PEC water splitting area.

## 2. Experimental methods

### 2.1. Materials

Bismuth nitrate pentahydrate (Bi(NO_3_)_3_·5H_2_O), acetic acid (CH_3_COOH), acetylacetone (C_5_H_8_O_2_), bis(acetylacetonato)dioxomolibdenum(VI) ([CH_3_COCH=C(O–)CH_3_]_2_MoO_2_), titanium isopropoxide (C_12_H_28_O_4_Ti), copper sulphate pentahydrate (CuSO_4_·5H_2_O), iron sulphate heptahydrate (FeSO_4_. 7H_2_O), sodium molybdate dihydrate (NaMoO_4_·2H_2_O) and *n*-methyl-2-pyrrolidone (NMP) (C_5_H_9_NO) was purchased from Sigma Aldrich. Vanadyl acetylacetonate (C_10_H_14_O_5_V), lactic acid (C_3_H_6_O_3_), sodium hydroxide (NaOH) were purchased from Avra chemicals. Thiourea (CH_4_N_2_S) from SDFCL limited. Fluorine doped tin oxide (FTO) substrates (surface resistivity ∼7 Ω sq.^−1^) were purchased from Sigma Aldrich. All the chemicals used in the study were of analytical grade and used without further purification.

### 2.2. Preparation of bismuth vanadate (BiVO_4_)/molybdenum doped BiVO_4_ (Mo-BiVO_4_) photoanode

FTO substrates were cleaned ultrasonically in acetone, isopropanol, ethanol and DI water respectively in a sequential manner for 5 minutes each. BiVO_4_ solution was prepared by adding a 0.173 g of bismuth nitrate pentahydrate and 0.097 g of vanadyl acetylacetonate in the mixture of 0.6 mL of acetic acid and 4.4 mL of acetylacetone. For Mo doping, 3 at% bis(acetylacetonato)dioxomolibdenum(vi) was added in the prepared BiVO_4_ solution.^33^ The prepared sol was magnetically stirred at 900 rpm for 1 hour to form a homogenous solution without precipitate. The homogeneous solution was spin coated at 1000 rpm for 30 seconds on FTO substrates. The spin coated substrates were annealed at 450 °C for 10 minutes. The same steps were repeated for four times to get an optimum performance. Finally, the substrates were annealed at 450 °C for 2 hours in air with rate of 5 °C min^−1^ in a muffle furnace.^34^

### 2.3. Preparation of cuprous oxide (Cu_2_O) photocathode

Cu_2_O photocathode was prepared by electrodeposition method using lactate stabilized copper sulphate solution.^35^ Initially, 0.4 M copper sulphate pentahydrate was mixed in 3 M lactic acid. The pH of the solution was changed to 10 by gradually adding 10 M NaOH. Electrodeposition process was carried out under a constant potential mode using three electrode setup in which FTO substrate, Ag/AgCl (Saturated KCl) and Pt wire used as a working electrode, a reference electrode and a counter electrode, respectively. A constant potential of −0.3 V *vs.* Ag/AgCl was applied for an hour to attain the desired thickness for effective optical absorption. After the deposition, the Cu_2_O coated FTO substrate was rinsed with DI water and dried at room temperature.

### 2.4. Deposition of TiO_2_ protective layer

The TiO_2_ protective layer was deposited on both BiVO_4_ and Cu_2_O photoelectrodes. Titanium isopropoxide and isopropanol was mixed in the volume ratio of 1 : 50 in a sample vial. The solution was sonicated for 15 minutes till a homogenous solution was formed without any visible chunks of titanium isopropoxide in the solution. The prepared solution was spin coated on the as-prepared photoanodes and photocathode at an optimized speed of 2000 rpm for 60 seconds. Only one layer was spin coated to maintain the minimum thickness for the protective layer. To improve the adhesivity of the TiO_2_ film, the photoelectrodes were annealed at 200 °C for 1 hour in air using a muffle furnace.^36^

### 2.5. Deposition of iron oxyhydroxide (FeOOH)

FeOOH cocatalyst was deposited on the as-prepared photoanodes electrochemically using three electrode setup in 0.1 M iron sulphate heptahydrate (FeSO_4_·7H_2_O) electrolyte solution. BiVO_4_, Ag/AgCl (sat'd KCl), and Pt were used as the working electrode, the reference electrode and the counter electrode, respectively. A constant potential of 1.2 V *vs.* Ag/AgCl was applied for a period of 300 seconds.^37^ After the deposition, the working electrode was rinsed in DI water and dried at room temperature.

### 2.6. Deposition of molybdenum disulphide (MoS_2_)

MoS_2_ catalyst was synthesized by hydrothermal method in which a 0.242 g of sodium molybdate dihydrate (Na_2_MoO_4_·2H_2_O) and 0.381 g of thiourea were mixed in 60 mL of DI water.^38^ The solution was then transferred to 100 mL Teflon-lined stainless steel autoclave. The autoclave was maintained at 200 °C for 24 hours. The obtained black precipitate was washed several times with DI water and ethanol followed by drying at 70 °C overnight.

For the deposition of MoS_2_ on Cu_2_O photocathode, 1 mg mL^−1^ of MoS_2_ powder was mixed with *n*-methyl-2-pyrrolidone (NMP) and sonicated for 3 hours. Then, the supernatant solution was taken and drop-casted on Cu_2_O photocathode followed by drying at 200 °C for 1 hour in air using a muffle furnace to improve the adhesion of MoS_2_ on Cu_2_O surface.

### 2.7. Photoelectrodes characterization

The structural characterization was performed using an X-ray diffractometer, D8 Advanced, Bruker with Cu kα radiation (*λ* = 1.5418 Å). The morphology of the photoanode was analysed using Field emission scanning electron microscope (FESEM), FEI Quanta 250 FEG. Optical characterization was carried out using UV-vis spectrometer, Specord Plus in the visible range. Vibrational characteristics was carried out using the Raman microscope, Horiba XploRA™ plus with 532 nm green laser as a source (25% laser power). X-ray photoelectron spectroscopy (XPS) was measured using PHI Versaprobe III. The obtained XPS spectra was fitted using CasaXPS software.

### 2.8. Photoelectrochemical characterization

Photoelectrochemical characterization was analysed using a 3-electrode setup in which photoanodes/photocathodes, Ag/AgCl (saturated KCl) and platinum wire were working, reference and counter electrodes, respectively. The illumination source was 300 W ozone-free Xenon lamp (Ushio, Japan) from Holmarc, India adjusted to the power intensity of 100 mW cm^−2^. The active area of the photoelectrodes was restricted to 1 cm^2^. The electrolyte used for all the PEC measurements was 0.1 M Na_2_SO_4_ (pH 6) and was purged with nitrogen for 30 minutes before the experiments. All the PEC measurements were recorded with respect to back-side illumination unless otherwise mentioned. A linear sweep voltammetry (LSV) was measured at a scan rate of 20 mV s^−1^. An electrochemical impedance spectroscopy (EIS) was performed under illumination at a frequency range of 10^5^ Hz to 1 Hz using an AC signal amplitude of 10 mV. Chronoamperometry measurement (stability measurements) of photoanode and photocathode was performed under chopped illumination at a potential of 1.23 V *vs.* RHE and 0 V *vs.* RHE for photoanode and photocathode, respectively for 2000 seconds. The potential of the working electrode was converted with respect to reversible hydrogen electrode (RHE) potential using the formula, *E*_RHE_ = *E*_Ag/AgCl_ + 0.059 pH + *E*^0^_Ag/AgCl_, where *E*_Ag/AgCl_ is the potential of working electrode with respect to Ag/AgCl and *E*^0^_Ag/AgCl_ is the standard potential of Ag/AgCl electrode which is 0.197 V.

### 2.9. Construction of tandem cell

Tandem cells were constructed with BiVO_4_ photoanode as the top electrode followed by Cu_2_O photocathode with a fixed distance of 1 cm. Upon illumination, light passed to BiVO_4_ photoanode (backside illumination) first and then unabsorbed light from BiVO_4_ transmitted to the Cu_2_O photocathode (front-side illumination). All tandem measurements were performed in 0.1 M Na_2_SO_4_ (pH 6) purged with nitrogen for 30 minutes before the experiments in a two electrode setup. The illuminated area of the cell was limited to 1 cm^2^. The linear sweep voltammetry (LSV) and the stability of the tandem cell was measured using a PARSTAT advanced electrochemical workstation. Stability tests of the tandem device was measured under zero bias condition.

## 3. Results and discussions

### 3.1. Structural characterization

**Fig. 1:**
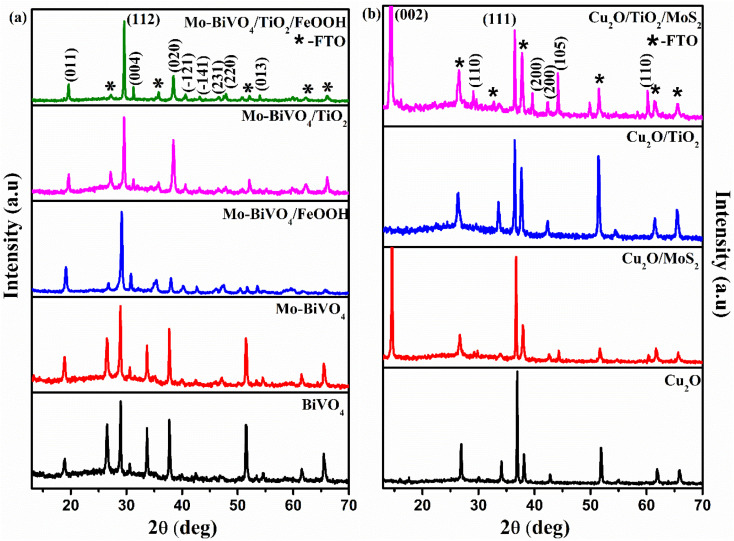
(a) X-ray diffraction spectra of BiVO_4_, Mo-BiVO_4_, Mo-BiVO_4_/FeOOH, Mo-BiVO_4_/TiO_2_ and Mo-BiVO_4_/TiO_2_/FeOOH photoanodes (b) X-ray diffraction spectra of Cu_2_O, Cu_2_O/MoS_2_, Cu_2_O/TiO_2_ and Cu_2_O/TiO_2_/MoS_2_ photocathodes.

The structural characterization of BiVO_4_ and modified BiVO_4_ photoanodes were analysed using XRD patterns. The XRD peaks of bare BiVO_4_ and the modified BiVO_4_ is presented in Fig. 1(a). The signature peaks at 18.8° and 28.9° in the photoanodes represent the monoclinic-scheelite structure of BiVO_4_.^39^ The results implied that the proper heat treatment of BiVO_4_ converts the tetragonal structure to monoclinic, which is preferred for better photocatalytic activity. The obtained results are well-matched to JCPDS card no. 014-6888 (ref. 40) and are indexed accordingly. No peaks related to Mo doping was observed due to minute atomic percentage of molybdenum^41^ but a slight red shift in the XRD spectra was observed for Mo doped BiVO_4_. The shift in the peak was due to the lattice strain caused by molybdenum on vanadium lattice points.^42^ Diffraction peaks of FeOOH was not found because of the amorphous nature of as-deposited co-catalyst. Similarly, a poor crystallization of the as-deposited TiO_2_ protective layer causes no diffraction peak. Similarly, XRD spectrum of Cu_2_O photocathodes is presented in Fig. 1(b). The obtained signature peaks at 36.8° and 43.5° for photocathodes represent [111] crystal plane orientation. All the obtained peaks corresponds to the JCPDS card no. 65-3288.^43^ The obtained XRD spectra of Cu_2_O photocathodes are cubic structure. The peak at 14.4° confirmed the presence of MoS_2_ in Cu_2_O/MoS_2_ and Cu_2_O/TiO_2_/MoS_2_ photocathodes.^44^ No peak was obtained for TiO_2_ because of its amorphous nature. No other extra peaks of copper oxide and metallic copper was observed in the obtained spectra. The obtained crystal structure of BiVO_4_ photoanodes and Cu_2_O photocathodes are monoclinic scheelite and cubic structures which provide better photocatalytic activity.

**Fig. 2:**
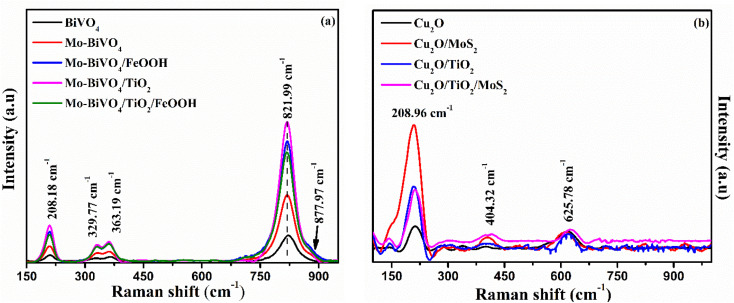
(a) Raman spectra of BiVO_4_, Mo-BiVO_4_, Mo-BiVO_4_/FeOOH, Mo-BiVO_4_/TiO_2_ and Mo-BiVO_4_/TiO_2_/FeOOH photoanodes and (b) Raman spectra of Cu_2_O, Cu_2_O/MoS_2_, Cu_2_O/TiO_2_ and Cu_2_O/TiO_2_/MoS_2_ photocathodes.

The Raman vibrational characteristics of BiVO_4_ photoanodes and Cu_2_O photocathodes are presented in Fig. 2(a) and (b). The peak at 208.18, 329.77, 363.19 and 822.3 cm^−1^ confirmed the vibrational spectra of BiVO_4_ and were assigned to external mode vibration of monoclinic BiVO_4_, symmetric and asymmetric deformation of VO_4_^3−^ and symmetric stretch mode of V–O, respectively. The BiVO_4_ peak at 821.99 cm^−1^ was blue shifted to 819.94 cm^−1^ for Mo-doped BiVO_4_ indicating the effect of Mo doping (Fig. S1(a) in the ESI†). A small hump observed at 885.5 cm^−1^ represented the bonding between molybdenum and oxygen present in BiVO_4_.^45^ No extra peaks were observed for TiO_2_ coating and FeOOH due to their amorphous nature. Similarly, Cu_2_O photocathodes exhibited a sharp Raman peak at ∼208.96 cm^−1^ which represents the second order Raman mode of Cu_2_O. The presence of MoS_2_ in Cu_2_O/MoS_2_ and Cu_2_O/TiO_2_/MoS_2_ photocathode was confirmed by doublet peaks at 376.65 cm^−1^ and 404.32 cm^−1^ (Fig. S1(b) in the ESI†). The peaks were assigned to in-plane and out-plane vibration of sulphur and molybdenum atoms.

### 3.2. Morphological characterization

**Fig. 3:**
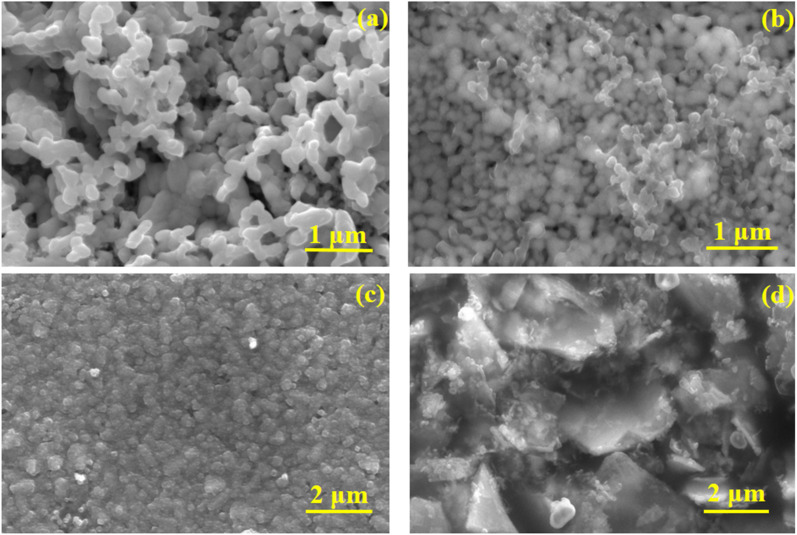
FESEM micrographs of (a) BiVO_4_ photoanode, (b) Mo-BiVO_4_/TiO_2_/FeOOH photoanode, (c) Cu_2_O photocathode, (d) Cu_2_O/TiO_2_/MoS_2_ photocathode.

The morphology of the as-prepared photoanodes and photocathodes were analysed using FESEM and the micrographs are presented in Fig. 3. The pure BiVO_4_ (Fig. 3(a)) photoanode exhibited nanoworm-like network morphology. No significant change in morphology was observed for molybdenum doped photoanode. The protective layer TiO_2_ was deposited on BiVO_4_ by cost effective spin coating method. The distribution of TiO_2_ confirmed by EDS mapping (Fig. S3 in the ESI†). No dramatic change in the morphology was observed for Mo-BiVO_4_/TiO_2_/FeOOH photoanode (Fig. 3(b)). The thickness of the Mo-BiVO_4_/TiO_2_/FeOOH photoanode layer was estimated to be *ca.* 706.9 nm from cross sectional FESEM micrograph (Fig. S2(a) in ESI†). The EDS mapping presented in Fig. S2† proves that all the layers are conformally coated on the photoanode. FESEM micrograph of Cu_2_O photocathode are presented in Fig. 3(c) and (d). The observed morphology of the photocathodes are nanospheres like structures (Fig. 3(c)). The MoS_2_ coating was found as sheets like structure throughout the photocathode (Fig. 3(d)). EDS mapping (Fig. S4 in the ESI†) confirms the presence of all layers on Cu_2_O photocathode. The thickness of the photocathode was estimated to be *ca.* ∼1.7 μm (Fig. S2(b) in ESI†) from the cross sectional FESEM micrograph. The EDS chemical elemental analysis of the Mo-BiVO_4_/TiO_2_/FeOOH photoanode and Cu_2_O/TiO_2_/MoS_2_ was further supported by the XPS analysis shown in Fig. S20 to S22 in the ESI.†

### 3.3. Optical characterization

**Fig. 4:**
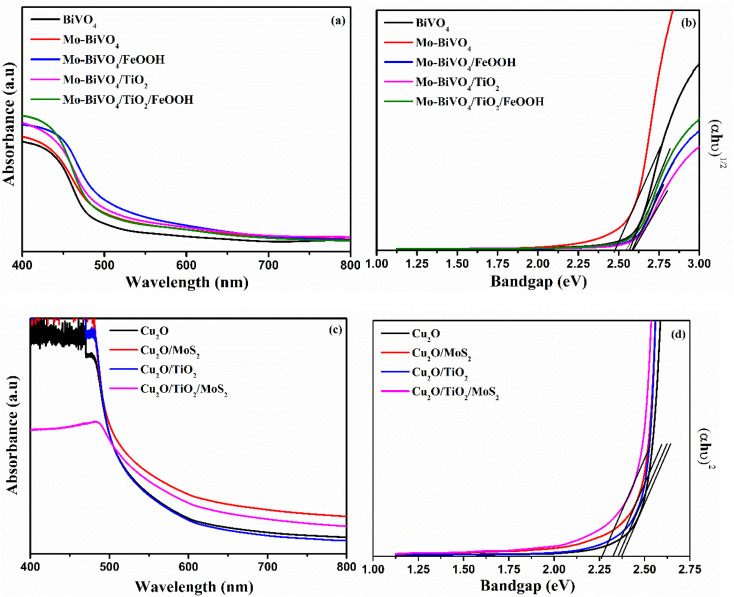
(a) UV-vis absorption spectra and (b) Tauc plot of BiVO_4_, Mo-BiVO_4_, Mo-BiVO_4_/FeOOH, Mo-BiVO_4_/TiO_2_ and Mo-BiVO_4_/TiO_2_/FeOOH photoanodes, (c) UV-vis absorption spectra and (d) Tauc plot of Cu_2_O, Cu_2_O/MoS_2_,Cu_2_O/TiO_2_, Cu_2_O/TiO_2_/MoS_2_ photocathodes.

The optical characterization of photoanode and photocathode was analysed by UV-vis spectrometer and the results are presented in Fig. 4. The absorbance spectra shows a typical optical absorption of BiVO_4_ with the absorption band edge starts from 500 to 550 nm for different composition of BiVO_4_ samples (Fig. 4 (a)). The optical bandgap was determined by constructing Tauc's plots (*αhν* = *k*(*hν* − *E*_g_)^*n*^) using an indirect optical transition (*n* = 2) for BiVO_4_. From the Tauc plot shown in Fig. 4(b), the calculated bandgap for bare BiVO_4_, Mo-BiVO_4_, Mo-BiVO_4_/FeOOH, Mo-BiVO_4_/TiO_2_, and Mo-BiVO_4_/TiO_2_/FeOOH was 2.54 eV, 2.47 eV, 2.57 eV, 2.59 eV and 2.56 eV respectively. The Cu_2_O photocathodes' optical absorption spectra and Tauc plot are shown in Fig. 4(c) and (d). The bandgap was calculated from Tauc plot using direct optical transition (*n* = ½). From the Tauc plot, the calculated bandgap for bare Cu_2_O, Cu_2_O/MoS_2_, Cu_2_O/TiO_2_, and Cu_2_O/TiO_2_/MoS_2_ was 2.37 eV, 2.32 eV, 2.35 eV and 2.26 eV, respectively.

### 3.4. Photoelectrochemical characterization

**Fig. 5:**
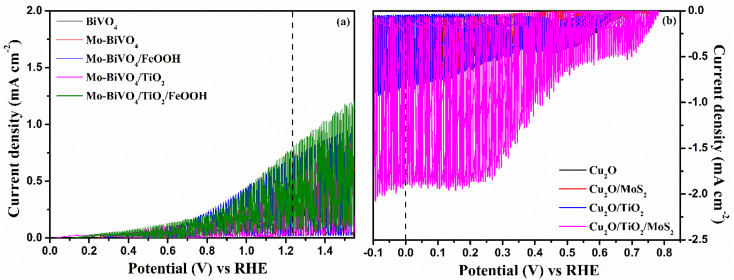
Chopped linear sweep voltammetry response of (a) BiVO_4_, Mo-BiVO_4_, Mo-BiVO_4_/FeOOH, Mo-BiVO_4_/TiO_2_ and Mo-BiVO_4_/TiO_2_/FeOOH photoanodes and (b) Cu_2_O, Cu_2_O/MoS_2_, Cu_2_O/TiO_2_ and Cu_2_O/TiO_2_/MoS_2_ photocathodes measured at 0.1 M Na_2_SO_4_ (pH 6) at 300 W Xenon lamp corrected to power intensity of 100 mW cm^−2^.

In order to confirm the effective operation of the tandem cell, individual photoelectrodes must be checked for the PEC activity. Hence, BiVO_4_ photoanodes and Cu_2_O photocathodes were tested in 3-electrode configuration under illumination. The photoelectrochemical behaviour of the BiVO_4_ photoanodes and Cu_2_O photocathodes was tested in 0.1 M Na_2_SO_4_ (pH 6). The chopped linear sweep voltammetry (LSV) of BiVO_4_ photoanode and Cu_2_O photocathode is presented in Fig. 5(a) and (b).

#### 3.4.1 Photoanode

The dark current density of all the photoanodes are almost negligible. The prepared photoanodes provided anodic response under illumination suggesting that the materials were of n-type (Fig. 5(a)). PEC parameters such as the onset potential and current density at water oxidation potential (1.23 V *vs.* RHE) are critical observation from LSV curves. There was a significant cathodic shift of onset potential observed when bare BiVO_4_ was doped with molybdenum and deposited with FeOOH and TiO_2_ layers. The results indicate that addition of dopants, co-catalysts and protective layer improved the conductivity, reduced the surface defects and enhanced interfacial charge transfer.^46^ The onset potential of bare BiVO_4_ estimated from the LSV curve was ∼0.55 V *vs.* RHE. The bare BiVO_4_ photoanode at water oxidation potential yielded a current density of ∼0.296 mA cm^−2^. The obtained current density was very low compared to the theoretical current density of BiVO_4_.^47^ Hence, modification on the surface of BiVO_4_ photoanode were performed. The onset potential of the Mo-BiVO_4_ was cathodically shifted to ∼0.40 V *vs.* RHE and an enhancement in the current density of ∼0.465 mA cm^−2^ was observed as a result of the improvement in the conductivity of the photoanode. In addition to doping, the TiO_2_ thin films and FeOOH co-catalysts were deposited to protect the surface and to reduce the overpotential of BiVO_4_, respectively. The photocurrent density of Mo-doped BiVO_4_/TiO_2_ photoanode was slightly better than Mo-doped BiVO_4_ but lower than Mo-doped BiVO_4_/FeOOH. As pointed out in the literature, a single overlayer on BiVO_4_ considerably minimizes the surface defects and as a consequence, the surface recombination is substantially suppressed compared to charge carrier kinetics.^48^ We assume that the TiO_2_ protective layer served its purposes as a protective layer to minimize surface defects. On the other hand, the Mo-BiVO_4_/FeOOH photoanode did the same protective role in addition to improve the reaction sites over TiO_2_ because of its catalytic nature. The trend in the increase in the current density was followed as: BiVO_4_ (∼0.296 mAcm^−2^) < Mo-BiVO_4_ (∼0.465 mAcm^−2^) < Mo-BiVO_4_/TiO_2_ (∼0.517 mA cm^−2^) < Mo-BiVO_4_/FeOOH (∼0.70 mA cm^−2^) < Mo-BiVO_4_/TiO_2_/FeOOH (∼0.81 mA cm^−2^). A four-fold increase in photocurrent was observed for Mo-BiVO_4_/TiO_2_/FeOOH compared to bare BiVO_4_ due to the deposition of 2 overlayers of which the TiO_2_ layer served as a protective layer and the FeOOH layer improved the kinetics of charge carriers. The stability test also suggested that the photoanode BiVO_4_/TiO_2_/FeOOH photoanode retained the current with a slight decay during the testing time of 2000 seconds (Fig. S7(a) in the ESI†). Overall, all the prepared photoanodes with an overlayer was stable during the testing time of which the Mo-BiVO_4_/TiO_2_/FeOOH outperformed all other samples as a result of a reduction in surface defects and an improvement in reaction kinetics. The comparison of Mo-BiVO_4_/TiO_2_/FeOOH photoanode and Cu_2_O/TiO_2_/MoS_2_ photocathode with other photoanodes and photocathodes and tandem structures reported in literature are tabulated in Tables S1, S2 and S7 in ESI,† respectively.

We also performed PEC tests of photoanodes in the presence of a hole scavenger (0.1 M Na_2_SO_4_ + 0.1 M Na_2_SO_3_) to measure the actual performance of the prepared photoanodes. To assess the actual performance of the prepared photoanodes, we calculated charge separation efficiency, injection efficiency and applied bias photon-to-current efficiency (ABPE). The results are shown in Fig. S8 in the ESI.† The photoanode coated with both the TiO_2_ protective layer and FeOOH co-catalyst outperformed all other prepared photoanodes in terms of charge separation, injection and ABPE. The charge separation efficiency, the amount of photogenerated charge reaching the surface of the photoelectrode, was calculated to be 62% (Fig. S8(b) in ESI†) for Mo-BiVO_4_/TiO_2_/FeOOH. The injection efficiency (Fig. S8(c) in ESI†) is the amount of photogenerated carriers reaching the surface and get injected into the solution. The Mo-BiVO_4_/TiO_2_/FeOOH photoanode exhibited ∼80% injection efficiency. The ABPE (Fig. S8(d)†) reached 0.16% at 0.9 V *vs.* RHE for Mo-BiVO_4_/TiO_2_/FeOOH photoanode. The Mo-BiVO_4_/TiO_2_ photoanode yielded better charge separation efficiency than the samples with Mo-BiVO_4_/FeOOH due to proper band level alignment of the former than the latter.^49^ On the other hand, the charge injection efficiency was better for Mo-BiVO_4_/FeOOH than the Mo-BiVO_4_/TiO_2_ photoanodes because of the reduction in overpotential generated by the FeOOH co-catalysts.^50^ Overall, the results suggest that both the protective and co-catalyst layers can yield better photoelectrochemical performances for BiVO_4_ photoanodes.

#### 3.4.2 Photocathodes

Similar PEC studies were conducted for Cu_2_O photocathodes to examine their photoelectrochemical properties. The LSV of Cu_2_O photocathodes is shown in Fig. 5(b). All Cu_2_O based photocathodes displayed cathodic response under illumination confirming the p-type nature of photocathodes. The current density of bare Cu_2_O reached to ∼−0.612 mA cm^−2^ at 0 V *vs.* RHE with an onset potential of 0.71 V *vs.* RHE. On the deposition of MoS_2_ catalyst, the current density increased to ∼−0.743 mA cm^−2^ at 0 V *vs.* RHE with an anodic shift in the onset potential of 0.75 V *vs.* RHE for Cu_2_O/MoS_2_ photocathode. The increase in current density could be an increase in the reaction sites provided by MoS_2_ co-catalysts for water reduction besides the protection of the Cu_2_O against photocorrosion. The photocurrent density of Cu_2_O/TiO_2_ photocathode showed an improved photocurrent of ∼−0.857 mA cm^−2^ than the Cu_2_O/MoS_2_ photocathode due to the conformal coating of TiO_2_. The results confirm that the spin-coated TiO_2_ protected layer effectively minimize photocorrosion and thereby enhanced the photocurrent. The maximum current density of ∼−1.880 mA cm^−2^ at 0 V *vs.* RHE with more anodic onset of 0.78 V *vs.* RHE was obtained for Cu_2_O/TiO_2_/MoS_2_ photocathode. The protection of Cu_2_O by TiO_2_ layer from photocorrosion and the improvement of the reaction kinetics by MoS_2_ catalysts enhanced the performance of the Cu_2_O/TiO_2_/MoS_2_ photocathode. The stability test showed that the Cu_2_O/TiO_2_/MoS_2_ photocathode sample was better stable during the testing window time of 2000 seconds without any significant decline in the photocurrent (Fig. S7(b) in ESI†). The photocurrent retained at ∼−1.36 mA cm^−2^ for Cu_2_O/TiO_2_/MoS_2_ photocathode during the testing time. On the other hand, the unprotected bare Cu_2_O photocathode's photocurrent declined quickly during the stability test due to instability of Cu_2_O photocathode in aqueous solution. The LSV response of best performing photoanode and photocathode was tested for front-side and back-side illumination and the response is presented Fig. S9 in the ESI.† The current density at water oxidation and reduction potentials, onset potential of BiVO_4_ photoanodes and Cu_2_O photocathodes are listed in Table 1.

Tabulation of photoelectrochemical performance of BiVO_4_ photoanodes and Cu_2_O photoanodes

**Table:** 

Photoanode	Onset potential (V) *vs.* RHE	Current density at 1.23 V *vs.* RHE (mA cm^−2^)
BiVO_4_	0.60	0.29
Mo-BiVO_4_	0.50	0.45
Mo-BiVO_4_/FeOOH	0.45	0.87
Mo-BiVO_4_/TiO_2_	0.23	0.54
Mo-BiVO_4_/TiO_2_/FeOOH	0.13	0.85

**Table:** 

Photocathode	Onset potential (V) *vs.* RHE	Current density at 0 V *vs.* RHE (mA cm^−2^)
Cu_2_O	0.37	−0.61
Cu_2_O/MoS_2_	0.78	−0.79
Cu_2_O/TiO_2_	0.64	−0.87
Cu_2_O/TiO_2_/MoS_2_	0.78	−1.88

#### 3.4.3 Electrochemical impedance spectroscopy (EIS) studies

**Fig. 6:**
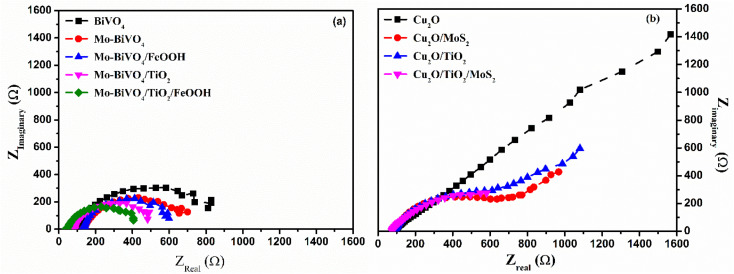
Electrochemical impedance spectra of (a) BiVO_4_, Mo-BiVO_4_, Mo-BiVO_4_/FeOOH, Mo-BiVO_4_/TiO_2_ and Mo-BiVO_4_/TiO_2_/FeOOH photoanodes measure at 1.23 V *vs.* RHE (b) Cu_2_O, Cu_2_O/MoS_2_, Cu_2_O/TiO_2_ and Cu_2_O/TiO_2_/MoS_2_ photocathodes measured at 0 V *vs.* RHE in 0.1 M Na_2_SO_4_ (pH 6) using 300 W Xenon lamp corrected to power intensity of 100 mW cm^−2^.

Electrochemical impedance spectroscopy (EIS) is a powerful tool to analyse the charge carrier kinetics of the photoelectrodes. The EIS experiment was carried out under illumination with respect to the water oxidation potential (1.23 V *vs.* RHE) and water reduction potential (0 V *vs.* RHE) for photoanode and photocathode, respectively. The EIS result of photoanode is shown in Fig. 6(a). As noticed from the spectra, it is obvious that the charge transfer resistance followed the order as: Mo-BiVO_4_/TiO_2_/FeOOH < Mo-BiVO_4_/TiO_2_< Mo-BiVO_4_/FeOOH < Mo-BiVO_4_ < BiVO_4_ from the shape of the semicircle. The obtained EIS spectra was fitted with different resistances and capacitances from which an equivalent circuit was constructed using Scribner's Z View software. The fitted values indicated a smaller charge transfer resistance for Mo-BiVO_4_/TiO_2_/FeOOH and thereby supported the maximum current obtained for this photoanode from the LSV response. The results also confirmed that the addition of both TiO_2_ and FeOOH minimized surface defects and also supported the findings of high charge injection efficiency and separation efficiency of the same photoanode. The EIS spectra of Cu_2_O photocathodes is shown in Fig. 6(b). The results revealed the charge transfer resistance in the following order: Cu_2_O/TiO_2_/MoS_2_ < Cu_2_O/TiO_2_ < Cu_2_O/MoS_2_ < Cu_2_O. The minimum *R*_s_ and *R*_ct_ was obtained for Cu_2_O/TiO_2_/MoS_2_ which was the best performing photocathode among Cu_2_O photocathodes as inferred from the LSV response. The results proved that the addition of TiO_2_ protective and MoS_2_ co-catalyst layers facilitate better charge transfer from the electrode to electrolyte. The equivalent circuit of photoanode and photocathode was similar to Randle's circuit and was presented in Fig. S10 in the ESI.† The fitted results of different resistances and capacitances for both photoanode and photocathode were tabulated in the ESI Table S5.† The Mott–Schottky analysis was performed in the dark condition at 1 kHz frequency for both BiVO_4_ based photoanodes and Cu_2_O based photocathodes. The corresponding Mott–Schottky plots of photoanodes and photocathode are illustrated in Fig. S11 and S12† and the flat band potential values are displayed in Table S6 in the ESI,† respectively.

#### 3.4.4 Tandem cell measurements

**Fig. 7:**
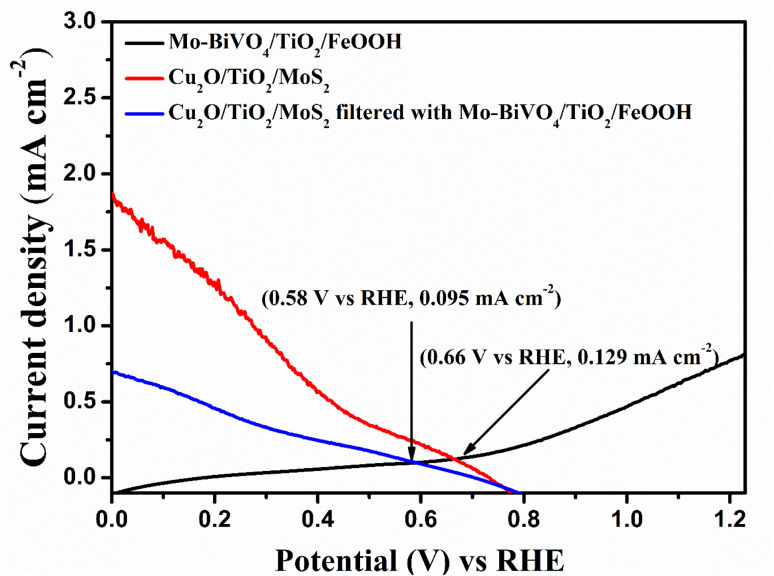
Overlaid LSV Plot of Mo-BiVO_4_/TiO_2_/FeOOH photoanode with Cu_2_O/TiO_2_/MoS_2_ photocathode and Cu_2_O/TiO_2_/MoS_2_ photocathode filtered by Mo-BiVO_4_/TiO_2_/FeOOH photoanode.

**Fig. 8:**
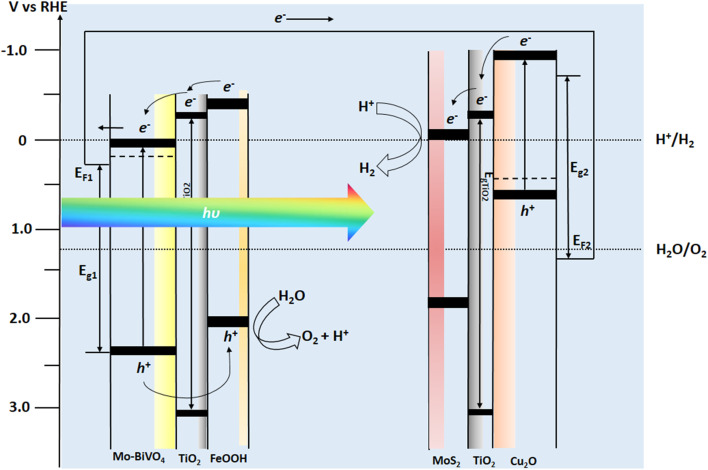
Energy band diagram of BiVO_4_–Cu_2_O tandem photoelectrochemical cell with respect to RHE potential.

After analysing the PEC properties of individual photoanode and photocathodes, we analysed overlaying the individual LSV response of the Mo-BiVO_4_/TiO_2_/FeOOH photoanode and Cu_2_O/TiO_2_/MoS_2_ photocathode as shown in Fig. 7. From the overlay plot, we obtained from the intersection point the upper limit of the operating potential (0.66 V *vs.* RHE) and operating current density (0.129 mA cm^−2^) for the tandem cell. The intersection point was also obtained for Cu_2_O/TiO_2_/MoS_2_ photocathode filtered with Mo-BiVO_4_/TiO_2_/FeOOH photoanode which were 0.58 V *vs.* RHE and 0.095 mA cm^−2^. The non-zero points provide the possibility of unassisted tandem cell operation. The energy band diagram of the proposed tandem cell which consisted of Mo-BiVO_4_/TiO_2_/FeOOH–Cu_2_O/TiO_2_/MoS_2_ is presented in Fig. 8. As noticed from the band diagram, the band positions are favoured for both the photoanode and photocathodes in terms of both electron and hole transports to the electrolyte for reduction and oxidation reactions, respectively.

**Fig. 9:**
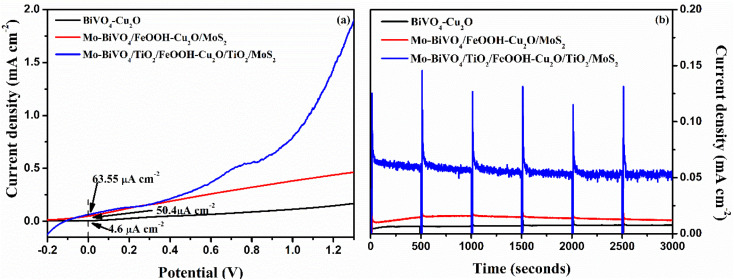
(a) 2-electrode LSV response of BiVO_4_-Cu_2_O, Mo-BiVO_4_/FeOOH-Cu_2_O/MoS_2_ and Mo-BiVO_4_/TiO_2_/FeOOH-Cu_2_O/TiO_2_/MoS_2_ tandem cells and (b) Unassisted stability test (*j vs. t*), (bias = 0 V) of BiVO_4_-Cu_2_O, Mo-BiVO_4_/FeOOH-Cu_2_O/MoS_2_ and Mo-BiVO_4_/TiO_2_/FeOOH-Cu_2_O/TiO_2_/MoS_2_ tandem cells measured in 0.1 M Na_2_SO_4_ (pH 6) using 300 W Xenon lamp corrected to power intensity of 100 mW cm^−2^.

The LSV response of the tandem cell was recorded from −0.2 to 1.3 V and the result is presented in Fig. 9(a). Compared to the performance of unprotected Mo-BiVO_4_/FeOOH-Cu_2_O/MoS_2_ tandem cell, the TiO_2_ protected tandem cell (Mo-BiVO_4_/TiO_2_/FeOOH-Cu_2_O/TiO_2_/MoS_2_) clearly shows an enhanced current density of +63.55 μA cm^−2^ at zero bias. On the other hand, the bare BiVO_4_–Cu_2_O tandem cell showed +4.6 μA cm^−2^ zero-bias photocurrent. In addition, we compared the performance of Mo-BiVO_4_/FeOOH-Cu_2_O/MoS_2_ tandem cell without TiO_2_ protective layer which produced +50.4 μA cm^−2^ at zero bias. The non-zero current density of +63.55 μA cm^−2^ at zero bias^51^ in 2-electrode LSV curve for Mo-BiVO_4_/TiO_2_/FeOOH– Cu_2_O/TiO_2_/MoS_2_ tandem cell (Fig. 9(a)) further proved the possibility of unassisted operation of tandem PEC cell.

The stability test of the tandem cell was conducted and the result is shown in Fig. 9(b). The stability curve of Mo-BiVO_4_/FeOOH–Cu_2_O/MoS_2_ was not stable for first 500 seconds and it started to decay for every 500 second cycle. On the other hand, the photocurrent stability of Mo-BiVO_4_/TiO_2_/FeOOH–Cu_2_O/TiO_2_/MoS_2_ produced a stable current density of ∼65.3 μA cm^−2^ with a slight decay at the end of 3000 seconds (∼53.9 μA cm^−2^). Despite the observed current density was low for TiO_2_ protected tandem structure, the photoelectrodes were very stable enough during the operating window of 3000 seconds. The results revealed that TiO_2_ deposited by spin coating method could be an alternative and economical approach to protect the photoelectrodes in aqueous environment.

### 3.5. Post-PEC analysis

Post-PEC analysis such as XRD (Fig. S14†), SEM with EDS (Fig. S15 to S19†), and XPS (Fig. S20 to S22†) was performed in order to understand the physical and chemical state of Mo-BiVO_4_/TiO_2_/FeOOH photoanode and Cu_2_O/TiO_2_/MoS_2_ photocathode (See ESI†). The crystallinity of the prepared photoelectrodes retained the same XRD spectra after the PEC tests suggesting that there is no change in the crystalline phase of the prepared photoelectrodes. From the EDS studies, we observed that the chemical state of co-catalyst FeOOH intensity was decreased and also the chemical state of MoS_2_ was significantly decreased indicating the poor adhesion of co-catalysts on the photoelectrode which will be improved in the future work. Similarly, XPS analysis also supported the EDS findings that there is a significant loss of both co-catalysts after the PEC test. In addition, we observed the presence of both Cu^+^ (Cu_2_O) and Cu^2+^ (CuO) peaks in the XPS spectra. The result indicates the possibility of CuO also in the sample after PEC test. The reason could be exposure of Cu_2_O on the side edges and/or the protective layer deposition may be not conformal in addition to some porosity. Despite the change in chemical state of copper, the TiO_2_ protected photocathodes performed better than the bare photoelectrodes. We will explore and optimize the deposition parameters of protective layer in our future work.

## 4. Conclusions

Thin films BiVO_4_ photoanode and Cu_2_O photocathode tandem cell protected by spin-coated TiO_2_ was demonstrated for unassisted solar water splitting. The photoanode consisted of Mo-BiVO_4_/TiO_2_/FeOOH yielded better photocurrent than bare BiVO_4_. The improvement in photocurrent for Mo-BiVO_4_/TiO_2_/FeOOH was attributed to (i) the TiO_2_ layer which protected the surface from defects and (ii) the FeOOH layer which improved the kinetics of charge carriers. The results were further supported by EIS spectra and charge separation and injection studies. The photocathode consisted of Cu_2_O/TiO_2_/MoS_2_ showed better PEC performance compared to bare Cu_2_O because of moderate protection of Cu_2_O from photocorrosion and an improvement of charge carrier kinetics by MoS_2_. The tandem cell made of Mo-BiVO_4_/TiO_2_/FeOOH–Cu_2_O/TiO_2_/MoS_2_ produced a stable current density of ∼65.3 μA cm^−2^ at zero bias with better stability and the retention percentage of photocurrent was 83.6% in the unassisted stability test. The co-catalyst dissolution after the PEC test due to improper adhesion will be further explored. The results suggested that spin-coated TiO_2_ could be an alternative viable approach for achieving moderately better PEC performance compared to unprotected photoelectrodes. The quality of TiO_2_ can be further explored by varying the spin speed, concentration, and annealing temperature to better optimize the films.

## Author contributions

Sitaaraman: methodology, writing-original draft. Nirmala Grace A: funding acquisition, validation. Raja Sellappan: methodology, writing and review – original draft, funding acquisition, validation.

## Conflicts of interest

The authors declare that they have no known competing financial interests or personal relationships that could have appeared to influence the work reported in this paper.

## Supplementary Material

RA-012-D2RA05774C-s001
