# Supplementary material for: Photoelectrochemical performance of a spin coated TiO2 protected BiVO4-Cu2O thin film tandem cell for unassisted solar water splitting
Source: RSC Adv. 2022 Nov 2;12(48):31380–91. doi: 10.1039/d2ra05774c (PMC9627459; doi:10.1039/d2ra05774c)
Supplement: RA-012-D2RA05774C-s001 [file RA-012-D2RA05774C-s001.pdf]

This journal is © The Royal Society of Chemistry 2022

Supplementary Information

Photoelectrochemical Performance of Spin Coated TiO_2 Protected $BiVO_4$ - Cu_2O Thin Films Tandem Cell for Unassisted Solar Water Splitting

S.R.Sitaaraman[1], A. Nirmala Grace[2], Raja Sellappan[2]

[1]School of Electronics Engineering, Vellore Institute of Technology, Vellore, India-632014.

[2]Centre for Nanotechnology Research, Vellore Institute of Technology, Vellore, India-632014.

1. Structural characterization

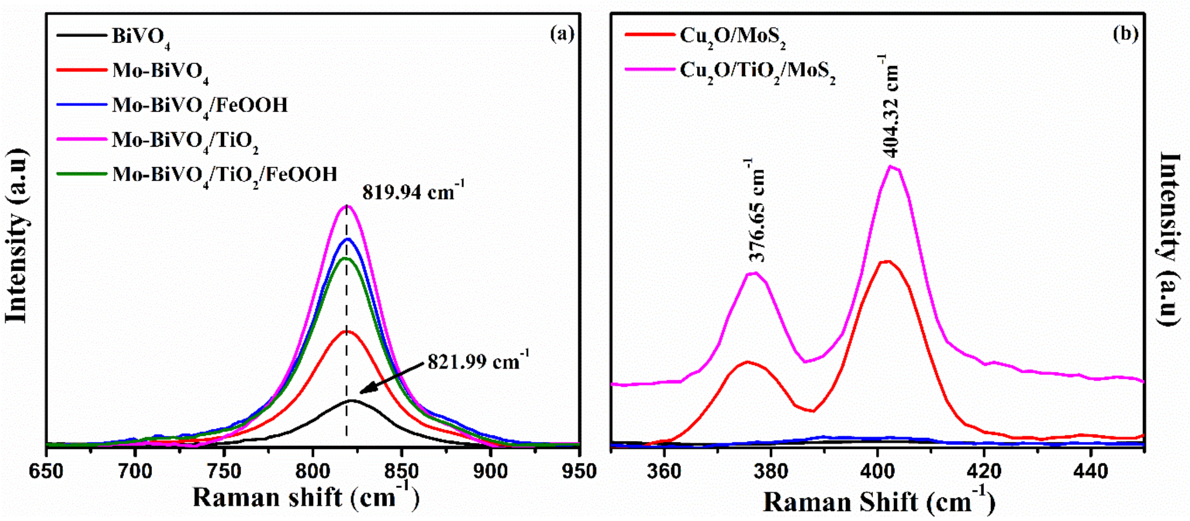

Figure S1, *Raman Spectra indicating the presence of Mo doping related peak in Mo-$BiVO_4$, Mo-$BiVO_4$/FeOOH, Mo-$BiVO_4$/TiO_2 and Mo-$BiVO_4$/TiO_2/FeOOH photoanodes and (b) Raman spectra indicating the presence of MoS_2 co-catalyst in Cu_2O/MoS_2 and Cu_2O/TiO_2/MoS_2 photocathodes*

2. Morphological characterisation

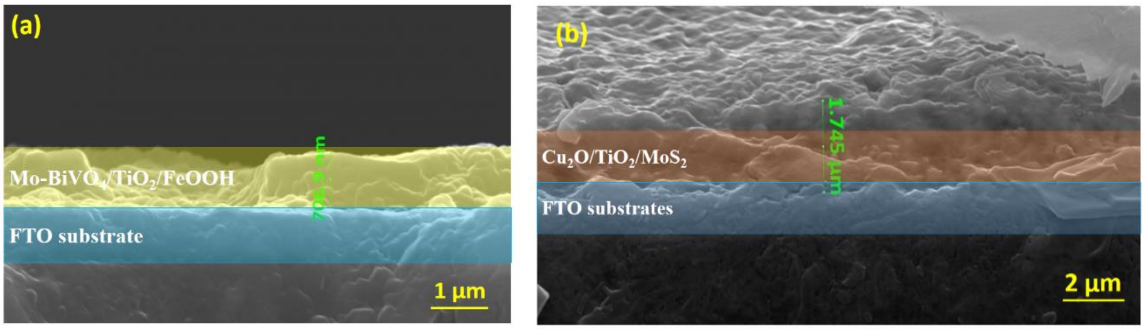

Figure S2, *Cross-sectional FESEM micrograph of (a) Mo-$BiVO_4$/TiO_2/FeOOH photoanode and (b) Cu_2O/TiO_2/MoS_2 photocathode*

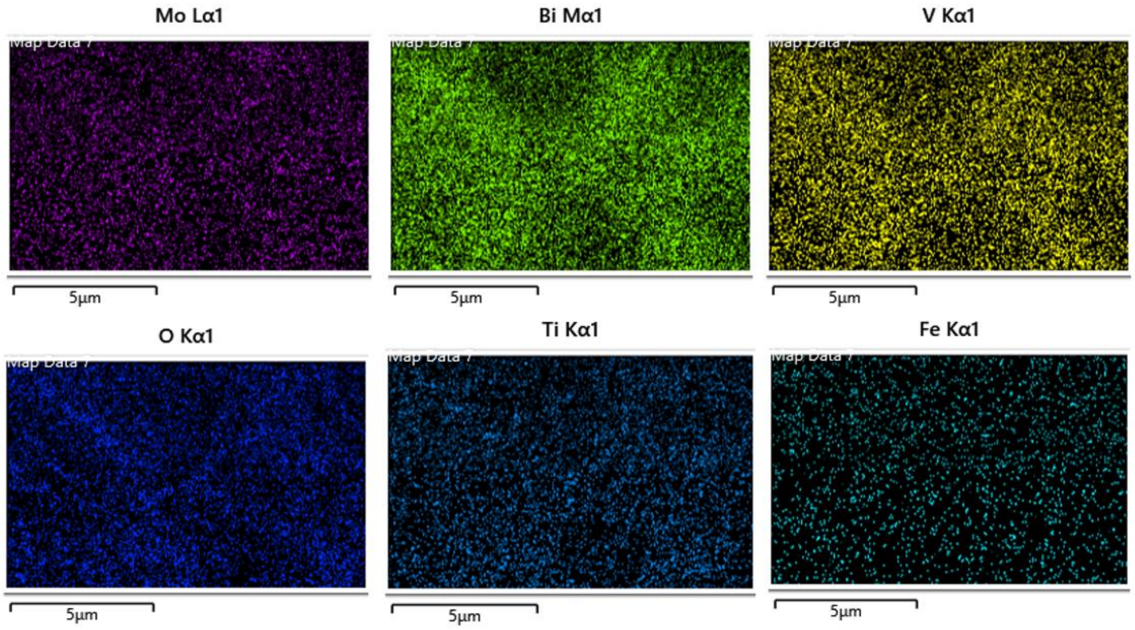

Figure S3, *EDS mapping spectra of Mo-BiVO$_4$/TiO$_2$/FeOOH photoanode*

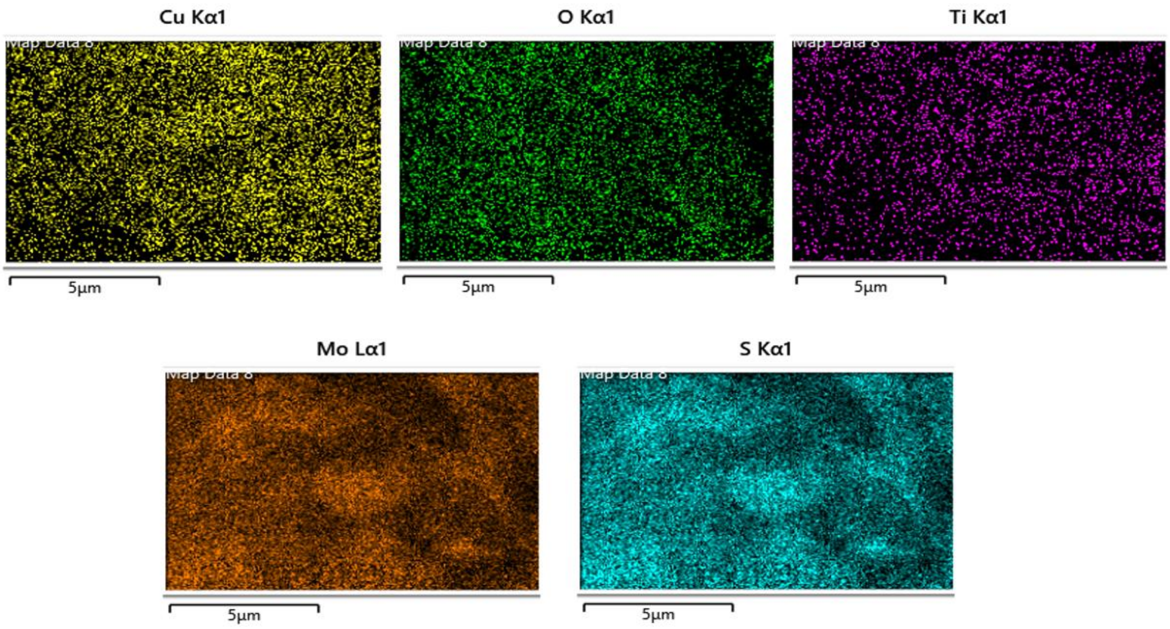

Figure S4, *EDS mapping spectra of Cu$_2$O/TiO$_2$/MoS$_2$ photocathode*

3. Photoelectrochemical characterization

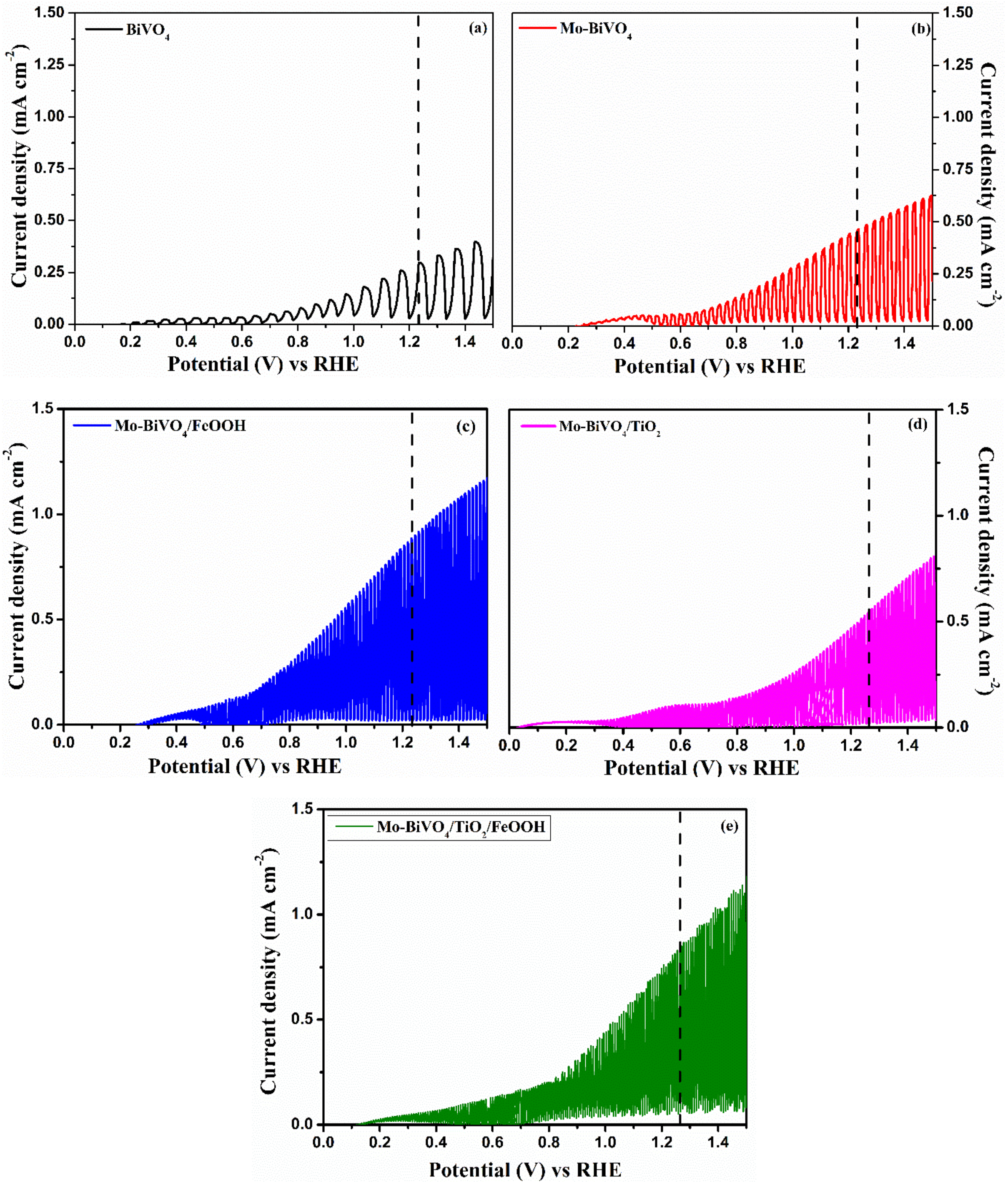

***Figure S5**, Chopped LSV response of (a) $BiVO_4$, (b) Mo-$BiVO_4$, (c) Mo-$BiVO_4$/FeOOH, (d) Mo-$BiVO_4$/TiO_2 and (e) Mo-$BiVO_4$/TiO_2/FeOOH photoanodes in 0.1 M Na_2SO_4 (pH 6) tested using 300 W Xenon lamp corrected to power intensity 100 mW cm^{-2}*

Table S1, *Comparison of various PEC performance of $BiVO_4$ based photoanodes reported in literature*

Photoanode	Preparation method	Electrolyte (pH)	Current density ($mA\ cm^{-2}$) at 1.23 V vs RHE	References
$BiVO_4/TiO_2$/FeOOH	Electrodeposition method	0.2 M phosphate buffer (pH 8)	3.28 mA cm^{-2}	1
W doped $BiVO_4$	Spray pyrolysis	0.5 M Na_2SO_4 + 0.09M KH_2PO_4	1.5 mA cm^{-2}	2
$BiVO_4$/NiO	Electrodeposition method	0.5 M Na_2SO_4 (pH 7)	1.2 mA cm^{-2}	3
$BiVO_4$/ Cu porphyrin	Electrodeposition method	0.1 M Na_2SO_4	1.25 mA cm^{-2}	4
Mo doped $BiVO_4$	Hydrothermal method	0.5 M Na_2SO_4	0.8 mA cm^{-2}	5
Li doped $BiVO_4$	Pulsed laser deposition	Phosphate buffer solution	0.8 mA cm^{-2}	6
$WO_3/BiVO_4$	Metal organic deposition method	0.1 mol/L phosphate buffer solution (pH 7.2)	0.9 mA cm^{-2}	7
$WO_3/BiVO_4$	Spin coating method	0.5 M Na_2SO_4	0.43 mA cm^{-2}	8
$BiVO_4$	Electrodeposition method	0.5 M Na_2SO_4 + 20 mM $Fe_2(SO_4)_3$ (pH 2.4)	1 mA cm^{-2}	9
W,Mo doped $BiVO_4$/FeOOH	Drop casting	0.1 M Na_2SO_4 (pH 7)	0.85 mA cm-2	10
W doped $BiVO_4$/FeCoW	Dip coating	1 M NaOH	0.6 mA cm^{-2}	11
Mo doped $BiVO_4/TiO_2$/FeOOH	Spin coating	0.1 M Na_2SO_4 (pH 6)	0.81 mA cm^{-2}	THIS WORK

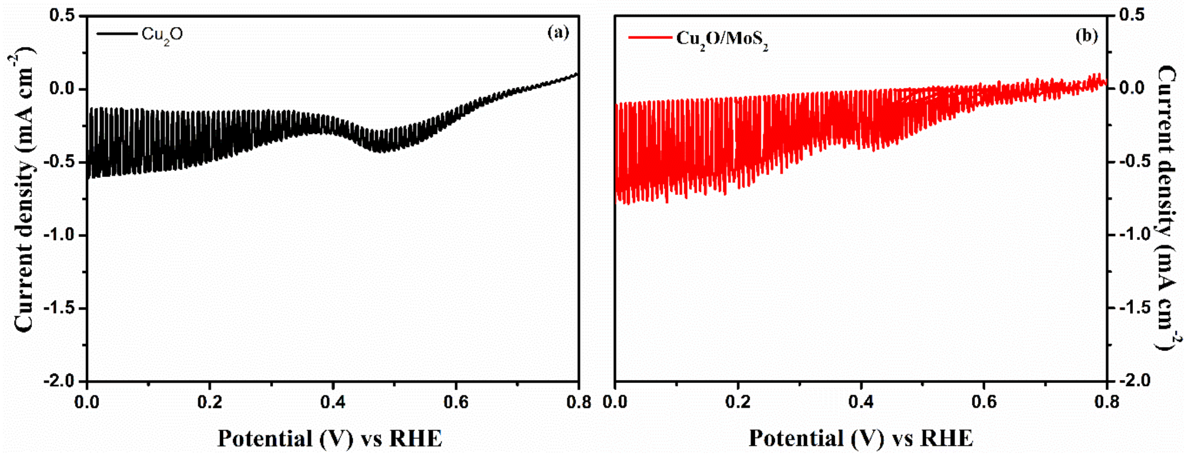

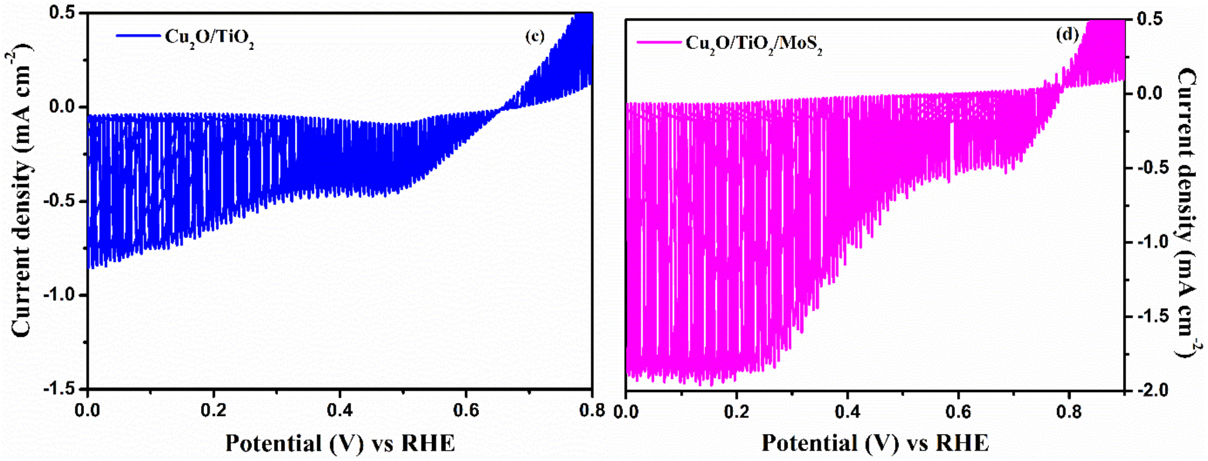

***Figure S6**, Chopped LSV response of (a) Cu_2O, (b) Cu_2O/MoS_2, (c) $Cu_2O/TiO_2/MoS_2$ and (d) $Cu_2O/TiO_2/MoS_2$ photocathodes 0.1 M Na_2SO_4 (pH 6) tested using 300 W Xenon lamp corrected to power intensity 100 mW cm^{-2}*

***Table S2,** Comparison of various PEC performance of Cu_2O based photoanodes reported in literature*

**Photocathode**	**Preparation method**	**Electrolyte (pH)**	**Current density (mA cm^{-2})**	**References**
$Cu/Cu_2O/AZO/TiO_2/RuO_x$	Electrodeposition method	0.5 M Na_2SO_4 +0.1 M phosphate buffer (pH 9)	-6.5 mA cm^{-2}	12
Cu_2O	Electrodeposition method	0.2 M phosphate buffer (pH 8)	-1.8 mA cm^{-2}	1
Cu_2O	Electrodeposition method	0.5 M Na-$_2SO_4$	-1.7 mA cm^{-2} at 0 V vs RHE	13
$Cu_2O/AZO/TiO_2/Pt$	Electrodeposition method	0.5 M Na_2SO_4	-1 mA cm^{-2} at 0 V vs RHE	14
Cu_2O nanoparticle	Wet chemical process	1 M Na_2SO_4	-0.2 mA cm^{-2} at -0.6 V vs Ag/AgCl	15
Cu_2O micro/nanostructures	Thermal decomposition method	0.5 M Na_2SO_4	-1.6 mA cm^{-2} at -0.6 V vs Ag/AgCl	16
Cu_2O/MoS_2	Hydrothermal method	0.5 M Na_2SO_4	-1.7 mA cm^{-2} at -0.6 V vs Ag/AgCl	17
Cu_2O/CuO	Electrodeposition method	0.1 M Na_2SO_4 (pH 6.25)	-1.2 mA cm^{-2} at 0 V vs RHE	18

Carbon modified Cu_2O nanoneedles	Thermal decomposition method	0.5 M Na_2SO_4 + 0.1 M KH_2PO_4 (pH 5)	-0.4 mA cm^{-2} at -0.5 V vs Ag/AgCl	19
$Cu_2O/NiO/Cu_2MoS_4$	Electrodeposition method	1 M Na_2SO_4 (pH 5)	-1.25 mA cm^{-2} at 0 V vs RHE	20
Cu_2O/NiFE LDH	Electrodeposition method	0.5 M Na_2SO_4	-0.4 mA cm^{-2} at -0.2 V vs Ag/AgCl	21
Au@Cu_2O core shell nanocrystals	Wet chemical process	0.5 M Na_2SO_4 (pH 6.5)	-30 µA cm^{-2} at 0.38 V vs RHE	22
$Cu_2O/TiO_2/MoS_2$	Electrodeposition method	0.1 M Na_2SO_4 (pH 6)	-1.88 mA cm^{-2} at 0 V vs RHE	THIS WORK

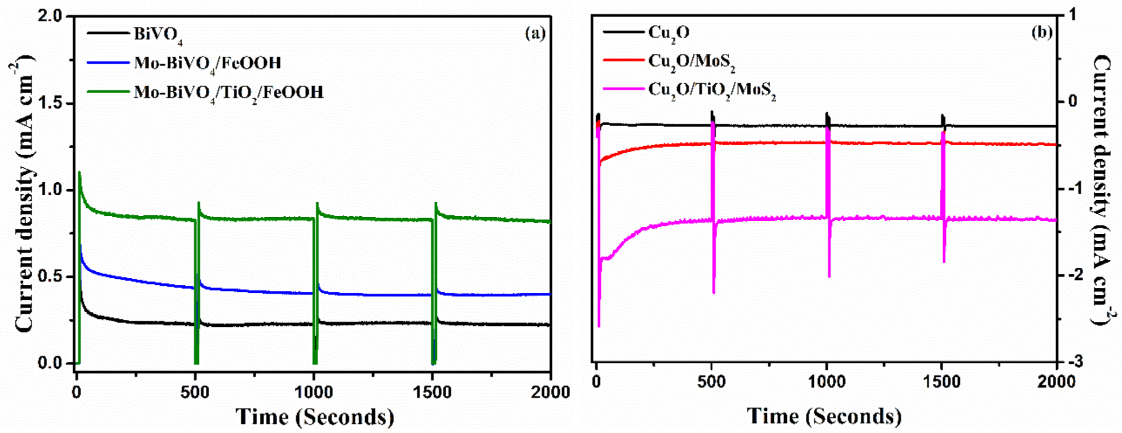

***Figure S7**, Chronoamperometric stability tests (j vs t) of (a) Bare $BiVO_4$, Mo-$BiVO_4$/FeOOH, and Mo-$BiVO_4/TiO_2$/FeOOH photoanodes at 1.23 V vs RHE (b) Bare Cu_2O, Cu_2O/MoS_2 and $Cu_2O/TiO_2/MoS_2$ photocathodes at 0 V vs RHE using 0.1 M Na_2SO_4 (pH 6) under 300 W Xenon lamp corrected to power intensity of 100 mW cm^{-2}. The illumination was chopped for every 500 seconds.*

The stability tests (j vs t) was performed for $BiVO_4$ photoanodes and Cu_2O photocathodes at 1.23 V vs RHE and 0 V vs RHE, respectively for 2000 seconds (figure S7). Light was not illuminated for the initial 10 seconds and the illumination was chopped for every 500 seconds. During dark condition, the photoanode show very low current density on the order of few µA cm^{-2}. When illumination was switched on after 10 seconds, a transient spike in current density was noticed due to the recombination effect. After some time, the current density remains stable for Mo-$BiVO_4/TiO_2$/FeOOH. The values of retention % of $BiVO_4$ photoanodes are tabulated in table S3. Similarly, Cu_2O, Cu_2O/MoS_2 and $Cu_2O/TiO_2/MoS_2$ photocathodes was tested at water reduction potential for 2000 seconds. Bare Cu_2O photocathode produces very low current density in the j vs t test. The addition of TiO_2 protective layer and MoS_2 co-catalyst increased

the stability and minimized the recombination by showing the retention percentage of 82.2 % and 84.4 % for Cu_2O/MoS_2 and $Cu_2O/TiO_2/MoS_2$ photocathode, respectively (Table S4).

Table S3, *Tabulation of retention percentage obtained from chronoamperometric stability tests (j vs t) (from figure S6 (b)) of* $BiVO_4$*, Mo-*$BiVO_4/FeOOH$ *and Mo-*$BiVO_4/TiO_2/FeOOH$ *photoanodes*

Photoanodes	Current density at 100 seconds (mA cm^{-2})	Current density at 2000 seconds (mA cm^{-2})	Retention (%)
**$BiVO_4$**	*0.268*	*0.223*	*83.2*
**Mo- $BiVO_4/FeOOH$**	*0.511*	*0.397*	*77.6*
**Mo- $BiVO_4/TiO_2/FeOOH$**	*0.867*	*0.826*	*95.2*

The retention percentage was calculated by dividing the current density obtained at 2000 s with the current density at 100 s.

Table S4, *Tabulation of retention percentage obtained from chronoamperometric stability test (j vs t) (from figure S6 (b)) of* Cu_2O/MoS_2 *and* $Cu_2O/TiO_2/MoS_2$ *photocathodes*

Photocathodes	Current density at 100 seconds (mA cm^{-2})	Current density at 2000 seconds (mA cm^{-2})	Retention (%)
**Cu_2O/MoS_2**	*-0.582*	*-0.488*	*82.2 %*
**$Cu_2O/TiO_2/MoS_2$**	*-1.637*	*-1.382*	*84.4 %*

Analysis of $BiVO_4$ based photoanodes in Na_2SO_3 hole scavenger

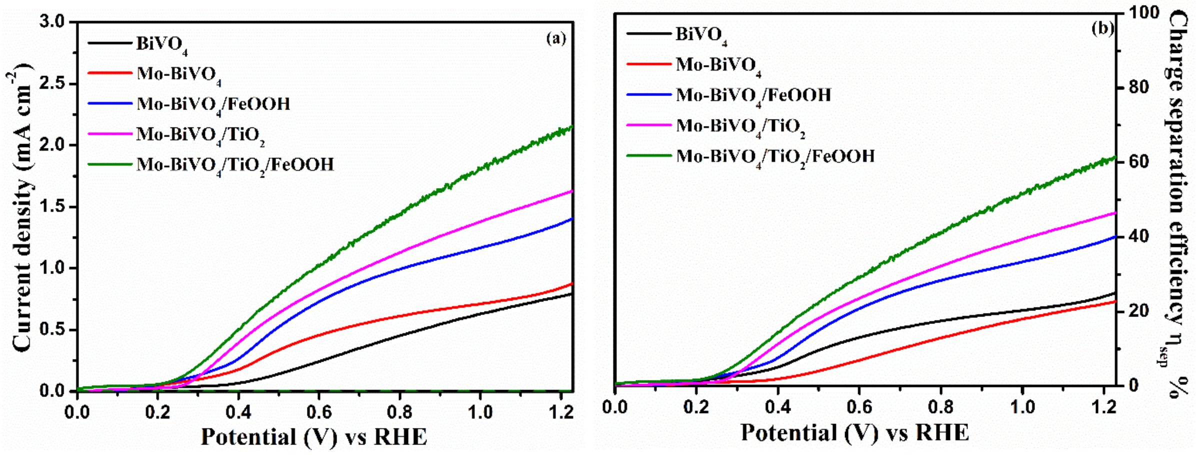

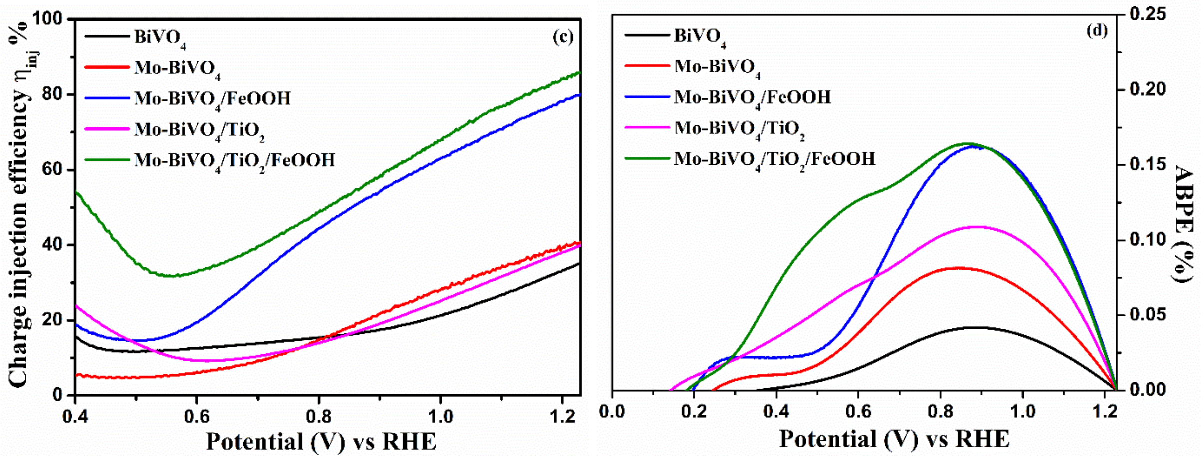

***Figure S8**, (a) LSV response of $BiVO_4$ photoanodes recorded in 0.1 M Na_2SO_4 + 0.1 M Na_2SO_3 hole scavenger, (b) Charge separation efficiency, (c) hole injection efficiency and (d) Calculated applied bias photon-to-current (ABPE) efficiency measured in 0.1 M Na_2SO_4 (pH 6) using 300 W Xenon lamp corrected to power intensity of 100 mW cm^{-2}*

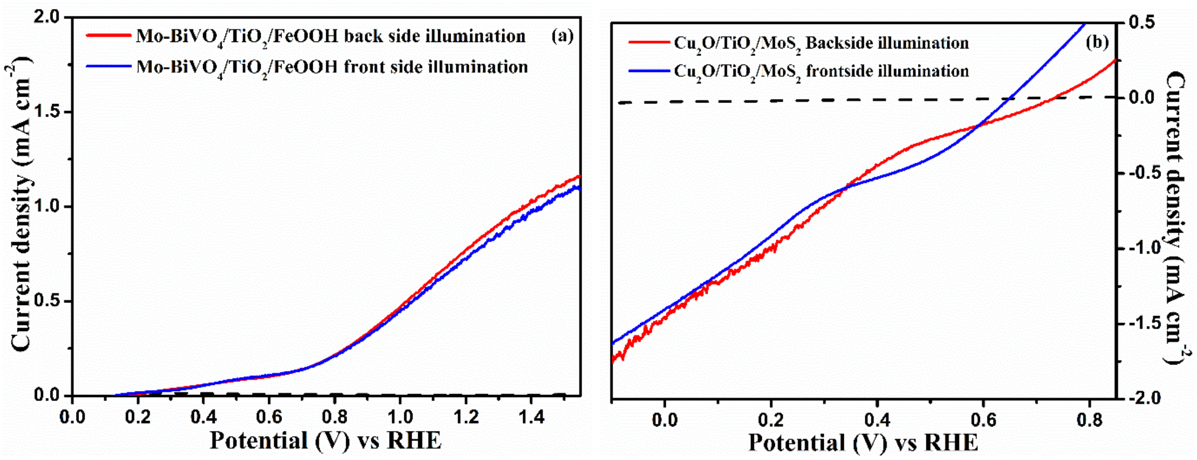

***Figure S9**, LSV response of front-side and back-side illumination of (a) Mo-$BiVO_4/TiO_2$/FeOOH photoanode and (b) $Cu_2O/TiO_2/MoS_2$ photocathode*

Electrochemical impedance spectroscopy studies

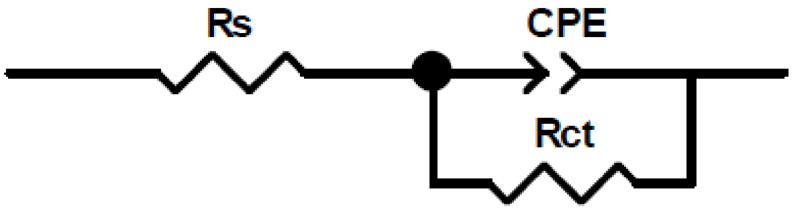

***Figure S10**, Equivalent circuit obtained by fitting EIS spectra of $BiVO_4$ photoanodes, Cu_2O photocathodes using Z View software. The equivalent circuit was obtained by using quick fit option and the equivalent circuit of all photoanodes and photocathodes is similar to Randle's circuit*

***Table S5**, Tabulation of obtained equivalent circuit parameters of all $BiVO_4$ photoanodes and Cu_2O photocathodes*

Photoanode	R_s (Ω)	R_{ct} (Ω)	CPE-T	CPE-P
**$BiVO_4$**	*361.7 Ω*	*934.9 Ω*	*6.457×10^{-5}*	*0.856*
**Mo-$BiVO_4$**	*134.2 Ω*	*610.3 Ω*	*7.101×10^{-5}*	*0.823*
**Mo-$BiVO_4$/FeOOH**	*130.5 Ω*	*499.7 Ω*	*6.4122×10^{-5}*	*0.892*
**Mo-$BiVO_4$/TiO_2**	*83.38 Ω*	*446.9 Ω*	*9.6228×10^{-5}*	*0.884*
**Mo-$BiVO_4$/TiO_2/FeOOH**	*41.83 Ω*	*416 Ω*	*0.00013607*	*0.827*
**Photocathode**	**R_s (Ω)**	**R_{ct} (Ω)**	**CPE-T**	**CPE-P**
**Cu_2O**	*89.79 Ω*	*1619 Ω*	*0.000199*	*0.48721*
**Cu_2O/MoS_2**	*75.65 Ω*	*812.5 Ω*	*3.067×10^{-5}*	*0.73933*
**Cu_2O/TiO_2**	*79.86 Ω*	*1091 Ω*	*5.807×10^{-5}*	*0.66102*
**Cu_2O/TiO_2/MoS_2**	*72.96 Ω*	*823.2 Ω*	*0.000234*	*0.69017*

Mott-Schottky analysis

$$\frac{1}{C_{sc}^2} = \frac{2}{\varepsilon_r \varepsilon_0 A^2 e N_{Dopant}} \left(E - E_{fb} - \frac{kT}{e}\right)$$

The Mott-Schottky plot is a measure of capacitance of space charge layer (C_{SC}) of the photoelectrodes as function of applied potential. where ε_r is the relative permittivity of the photoelectrode, ε_0 is the permittivity in vacuum, A is the surface area of the electrode, *e* is the charge of an electron, N_{Dopant} is the free carrier density, *k* is the Boltzmann constant, T is the temperature and E is applied potential.

The Mott Schottky plot of photoanodes and photocathodes were recorded in dark condition at 1 kHz frequency. The result of M-S plot is presented in figure S11 and S12. From the M-S plot, the type of the semiconductor material, flat band potential (E_{FB}) and donor density can be found which is paramount for analysing photoelectrochemical behaviour of the photoelectrodes. For $BiVO_4$ photoanodes (figure S11), the slope of the curve indicated the n-type behaviour. The x-intercept of the curve provides the E_{FB}. The variation in the flat band potential of $BiVO_4$ based photoanodes is little with respect to the addition of layers. The E_{FB} value of bare $BiVO_4$ was similar to the reported values in the literature [23] that is, ~ 0.048 V vs RHE. The major shift in the M-S plot was observed after the addition of TiO_2 protective layer and FeOOH catalyst. The E_{FB} was cathodically shifted to ~ -0.164V vs RHE for Mo-$BiVO_4$/TiO_2/FeOOH. The negative shift indicates the improvement in the overpotential of the water oxidation. The addition of FeOOH improves current density at a minimum applied potentials which in turn improves the overall efficiency of the tandem cell by increasing the operating points of the tandem cell. The Cu_2O based photocathodes showed anodic slope indicating p-type behaviour (figure S12). The

E_{FB} of Cu_2O based photocathodes was calculated using the corresponding x-intercept. The observed E_{FB} of the bare Cu_2O was 0.52 V vs RHE which is close to the reported value. The E_{FB} of $Cu_2O/TiO_2/MoS_2$ photocathode was shifted cathodically to 0.64 V vs RHE. The shift in the E_{FB} is attributed to the addition of TiO_2 protective layer as well as MoS_2 catalyst. The table S6 shows the measured flat band potential and the dopant density of all the prepared photoanodes and photocathodes. The more cathodic flat band values of Mo-$BiVO_4/TiO_2$/FeOOH among all the prepared photoanodes and the more anodic flat band values of $Cu_2O/TiO_2/MoS_2$ among all the prepared photocathodes could be better suitable for tandem cells as it can improve the current density at lower potentials due to reduced charge carrier recombination.

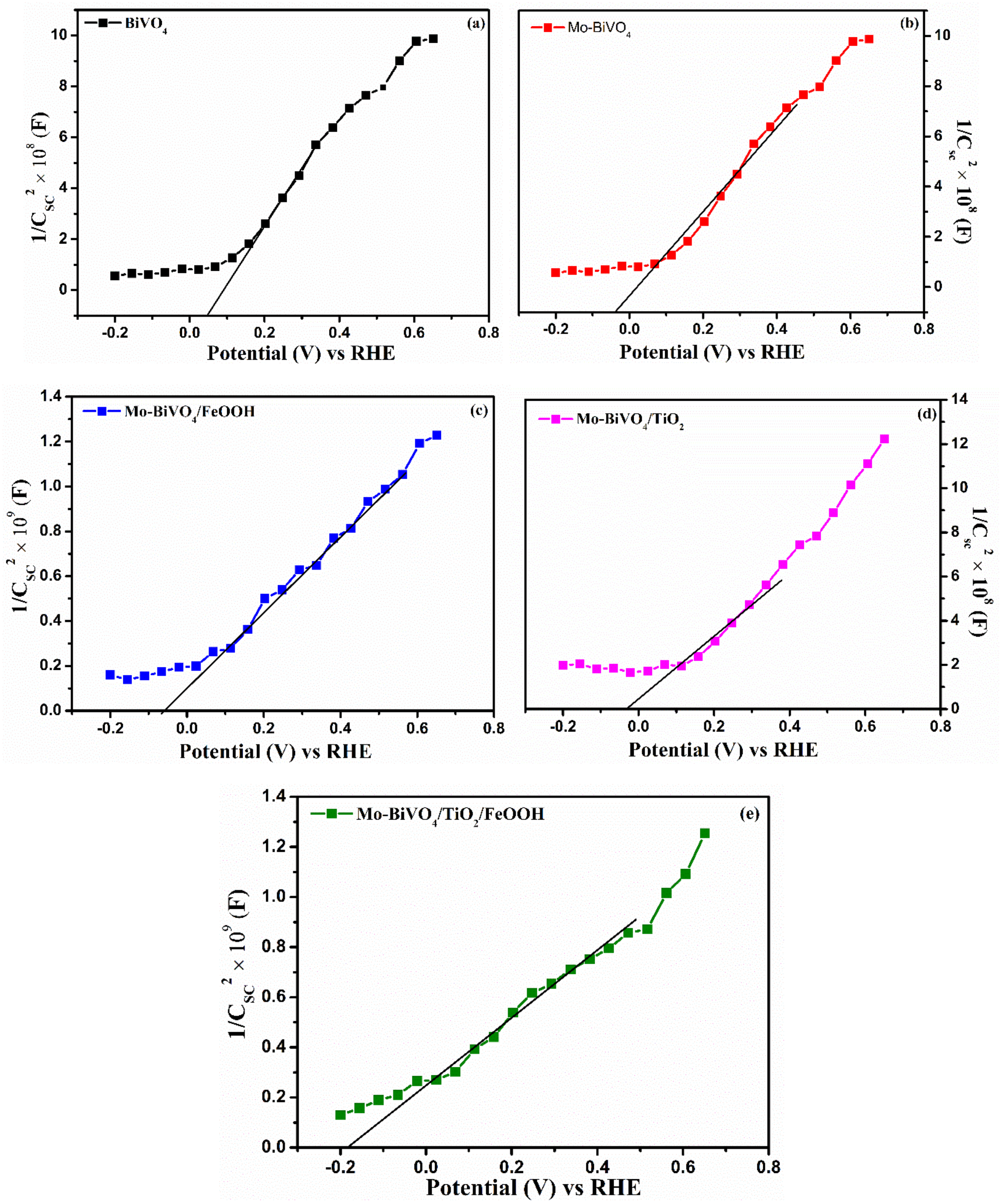

Figure S11*, Mott-Schottky plots of (a)$BiVO_4$, (b) Mo-$BiVO_4$, (c) Mo-$BiVO_4$/FeOOH, (d) Mo-$BiVO_4$/TiO_2, Mo-$BiVO_4$/TiO_2/FeOOH photoanodes measured in 0.1 M Na_2SO_4 (pH 6) under dark condition*

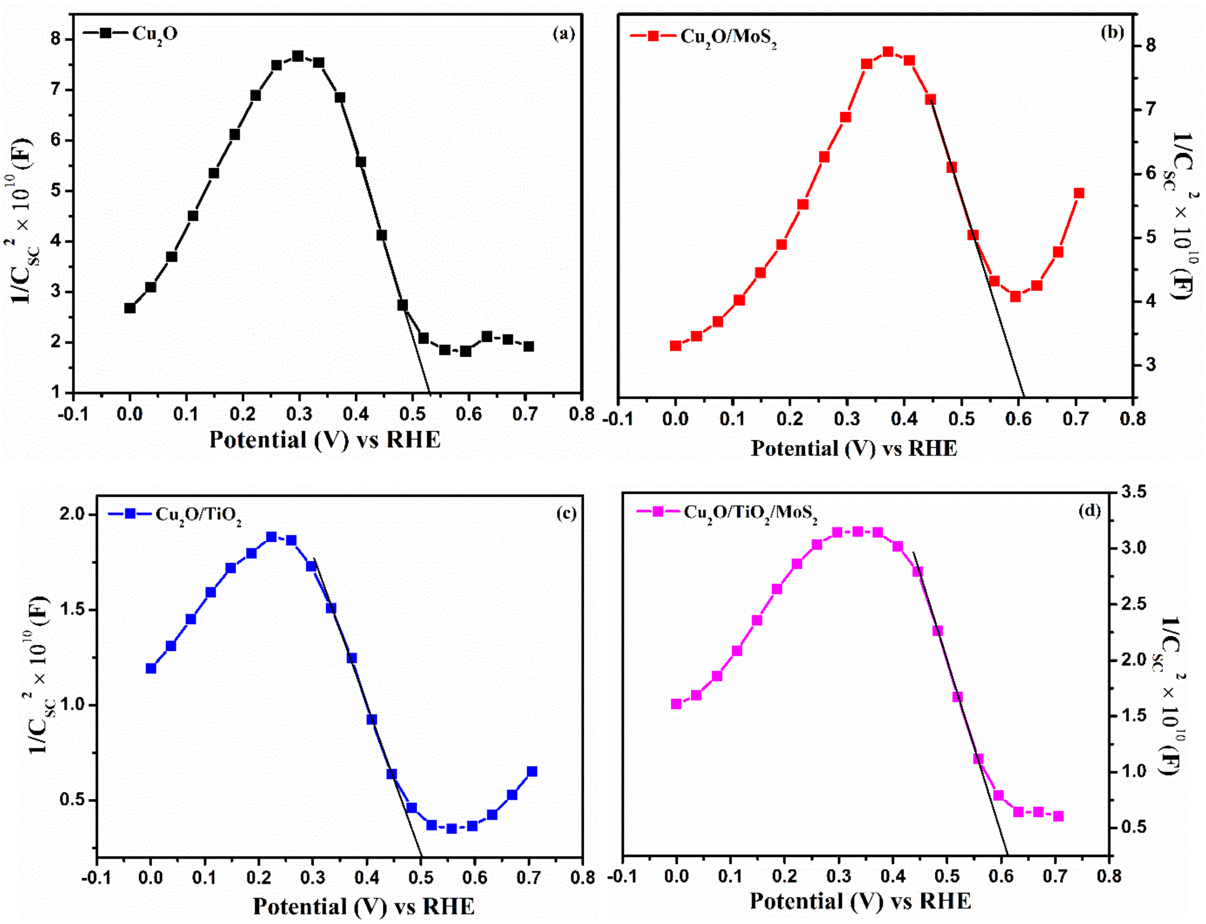

Figure S12*, Mott-Schottky plots of (a) Cu_2O, (b) Cu_2O/MoS_2, (c) Cu_2O/TiO_2, (d) $Cu_2O/TiO_2/MoS_2$ photocathodes measured in 0.1 M Na_2SO_4 (pH 6) measured under dark condition*

Table S6*, Tabulation of obtained parameters from Mott-Schottky plot of $BiVO_4$ photoanodes and Cu_2O photocathodes*

Photoanode	Flat band potential E_{FB} (V) vs RHE	Slope	Dopant density (cm^{-3})
**$BiVO_4$**	*-0.038 V*	*1.77×10^9*	*7.96×10^{21}*
**Mo-$BiVO_4$**	*-0.039 V*	*1.66×10^9*	*8.49×10^{21}*
**Mo-$BiVO_4$/FeOOH**	*-0.087 V*	*1.63×10^9*	*8.65×10^{21}*

**Mo-$BiVO_4/TiO_2$**	*-0.034 V*	*1.55×10^{9}*	*9.09×10^{21}*
**Mo-$BiVO_4/TiO_2$/FeOOH**	*-0.175 V*	*1.34×10^{9}*	*10.5×10^{21}*
**Photocathode**	**Flat band potential E_{FB} (V) vs RHE**	**Slope**	**Dopant density (cm^{-3})**
**Cu_2O**	*0.53*	*-3.32×10^{10}*	*4.24×10^{20}*
**Cu_2O/MoS_2**	*0.60*	*-2.43×10^{10}*	*5.80×10^{20}*
**Cu_2O/TiO_2**	*0.50*	*-7.13×10^{10}*	*1.97×10^{20}*
**$Cu_2O/TiO_2/MoS_2$**	*0.61*	*-1.38×10^{10}*	*10.2×10^{20}*

Constructed Tandem cell Images

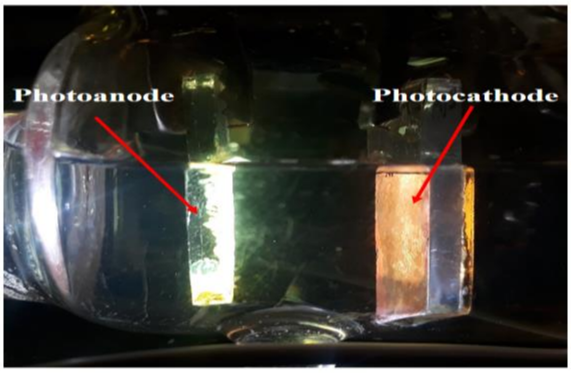

Figure S13, *Photograph of the constructed tandem cell (Mo-$BiVO_4/TiO_2$/FeOOH-$Cu_2O/TiO_2/MoS_2$)*

Table S7, *Comparison of reported $BiVO_4$ based tandem cells for unassisted solar water splitting*

**Photoanode**	**Photocathode**	**Operating points**	**Unassisted current density ($mA\ cm^{-2}$)**	**Electrolyte (pH)**	**Ref.**
W:$BiVO_4$/CoPi	$CuBi_2O_4$/CdS/TiO_2/RuO_x	0.3 mA cm^{-2}, 0.64 V vs RHE	0.1 mA cm^{-2}	0.3 M K_2SO_4 + 0.2 M phosphate buffer (pH 6.8)	[24]

H:$BiVO_4$/ $NiFeO_x$	Cu_2O/Ga_2O_3/ TiO_2/NiMo	2.45 mA cm^{-2}, 0.6 V vs RHE	2.5 mA cm^{-2}	0.1 M Na_2CO_3 +0.1 M $NaHCO_3$ (pH 9)	[12]
$BiVO_4$ nanostructures	Cu_2O/ H:$Ti_3C_2T_x$	0.45 mA cm^{-2}	0.28 mA cm^{-2}	0.1 M KPi (pH 7)	[25]
$BiVO_4$ nanostructures/ Co-B_i	$CuBi_2O_4$/ Co-B_i	0.11 mA cm^{-2}, 0.2 V vs RHE	36 µA cm^{-2}	0.5 M Na_2SO_4 (pH 9.2)	[26]
Mo doped $BiVO_4$/Co-Pi	$CuBi_2O_4$/Pt	0.15 mA cm^{-2}	0.1 mA cm^{-2}	0.1 M potassium phosphate (KPi) buffer	[27]
$BiVO_4$	CIGS/CdS/ Al_2O_3/TiO_2/ Pt	1 mA cm^{-2}	0.8 mA cm^{-2}	1 M Potassium borate (pH 9.2)	[28]
Mo-$BiVO_4$/Co-Pi	Si/Pt	0.6 mA cm^{-2}, 0.38 V vs RHE	0.15 mA cm^{-2}	0.1 M potassium phosphate (pH 5.5)	[29]
$BiVO_4/TiO_2$/FeOOH	Cu_2O	0.36 mA cm^{-2}, 0.37 V vs RHE	0.1 mA cm^{-2}	0.2 M phosphate buffer (pH 8)	[1]
$BiVO_4$	CZTS/HfO_2/ CdS/Pt	2.5 mA cm^{-2}, 0.6 V vs RHE	2.3 mA cm^{-2}	0.2 M phosphate buffer (pH 6.5)	[30]
W:$BiVO_4$	Si/Pt	2.5 mA cm^{-2} 0.55 V vs RHE	2.5 mA cm^{-2}	0.1 M potassium phosphate (pH 7.3)	[31]
W:$BiVO_4$	Cu_2O/AZO/ TiO_2/RuO_x	0.6 mA cm^{-2}, 0.45 V vs RHE	-0.1 mA cm^{-2}	0.5M Na_2SO_4 + 0.01 M K_2HPO_4	[2]
Mo-$BiVO_4/TiO_2$/FeOOH	Cu_2O/TiO_2/ MoS_2	0.129 mA cm^{-2}, 0.66 V vs RHE	65.3 µA cm^{-2}	0.1 M Na_2SO_4 (pH 6)	THIS WORK

4. Characterization of photoanodes and photocathodes after PEC test

4.1 Structural characterization

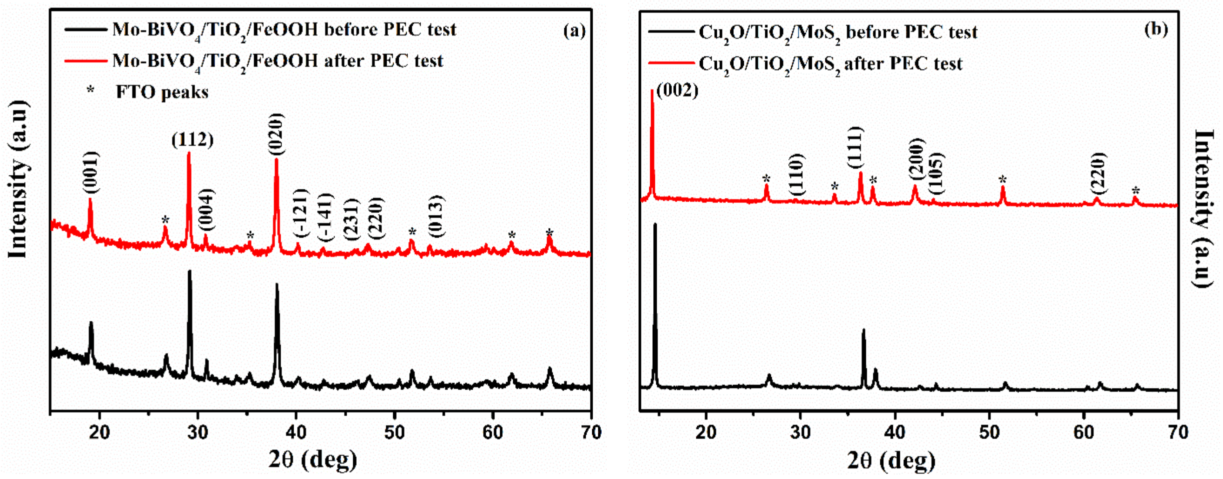

Figure S14, *XRD spectra of (a) Mo-BiVO$_4$/TiO$_2$/FeOOH photoanode and (b) Cu$_2$O/TiO$_2$/MoS$_2$ photocathode before and after PEC tests*

XRD spectra of Mo-BiVO$_4$/TiO$_2$/FeOOH photoanode and Cu$_2$O/TiO$_2$/MoS$_2$ photocathode is presented in figure S14. After conducting PEC performances such as LSV, j vs t (Stability), EIS and Mott-Schottky analysis were conducted on the photoanode and photocathode, the XRD spectra was again recorded. The XRD spectra of Mo-BiVO$_4$/TiO$_2$/FeOOH before and after PEC test (figure S14 (a)) are matched and well-indexed with JCPDS card no. 014-6888 [32]. The peak at 18° and 29° corresponds to the signature peak of monoclinic scheelite BiVO$_4$. No significant peaks was observed for TiO$_2$ and FeOOH catalyst layers. Similarly, after the PEC tests, no shift in peak was observed in the XRD spectra of Mo-BiVO$_4$/TiO$_2$/FeOOH photoanodes. Similarly, XRD spectra of Cu$_2$O/TiO$_2$/MoS$_2$ photocathode before and after PEC test (figure S14 (b)) corresponds to JCPDS card no.: 65-3288 [33]. The majority peak at 36.3° corresponds to the formation of Cu$_2$O (111) orientation. No shift or additional peaks obtained for TiO$_2$ layer because of the lower content of TiO$_2$. MoS$_2$ catalyst coated on the photocathode produced a major peak at 14.1°, 43.2° and 60.11° correspond to JCPDS card no.: 96-101-1287. Even after the PEC tests, the XRD spectra of photocathode matches well with the above mentioned JCPDS cards indicating no change in crystal phase.

4.2 Morphological characterization

FESEM micrograph of Mo-BiVO$_4$/TiO$_2$/FeOOH photoanode and Cu$_2$O/TiO$_2$/MoS$_2$ photocathode are presented in figure S15 (a to d). The Figure S15 (a) and (b) are the FESEM micrograph of Mo-BiVO$_4$/TiO$_2$/FeOOH before and after PEC test, respectively. The translucent film observed on top (S15 a) was attributed to FeOOH film which was further confirmed by EDS mapping shown in figure S16 and S17. After PEC test, the FeOOH layer on the photoanode was reduced due to improper adhesion and/or the prolonged exposure of the catalyst to the electrolyte. Figure S15 (c) and (d) show the FESEM micrograph of Cu$_2$O/TiO$_2$/MoS$_2$ before and after PEC test, respectively. The morphology of the photocathode before PEC tests was nano-sheets like morphology which was assigned to MoS$_2$ co-catalyst

layer. After PEC tests, the MoS_2 co-catalyst was reduced from the photocathode as evidenced from the EDS mapping presented in figure S18 and S19.

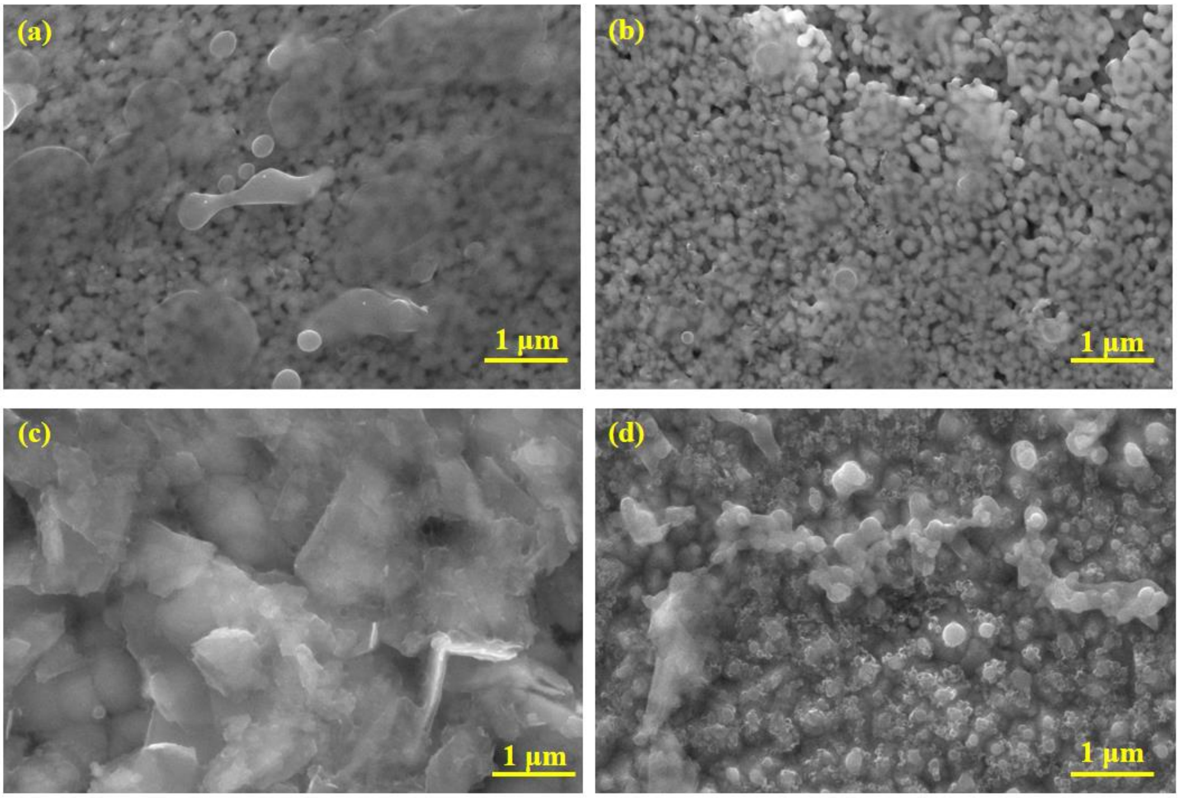

Figure S15, *FESEM micrograph of Mo-$BiVO_4$/TiO_2/FeOOH photoanode and Cu_2O/TiO_2/MoS_2 photocathode (a and c) before and (b and d) after PEC test*

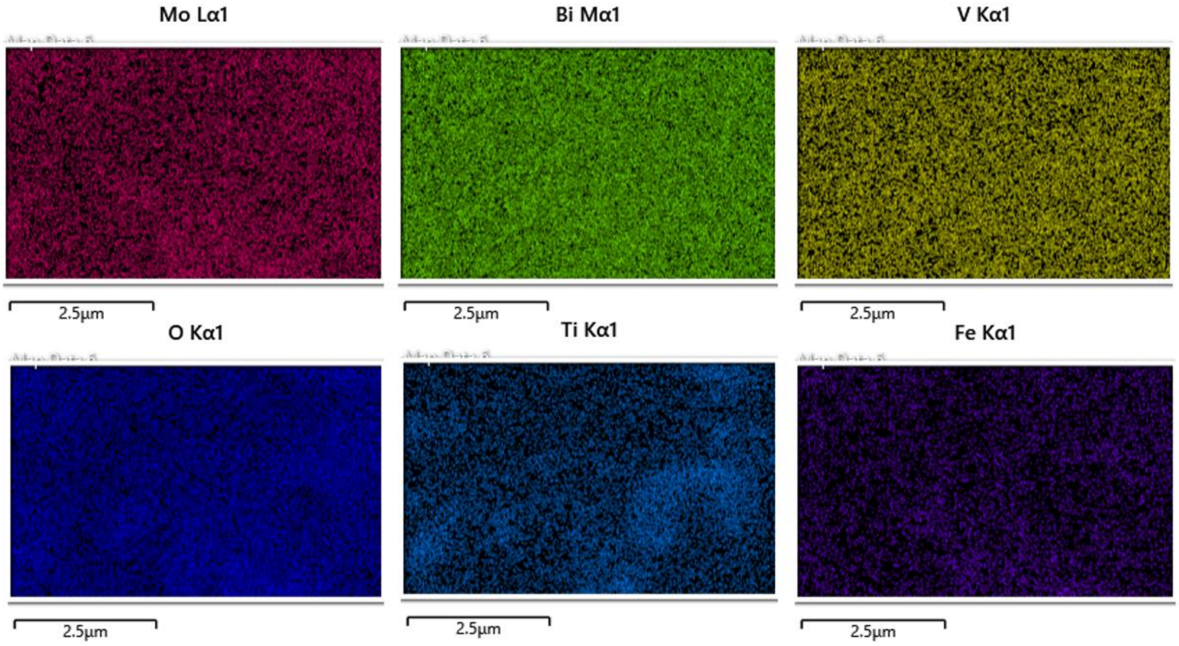

Figure S16, *EDS Colour mapping of Mo-$BiVO_4$/TiO_2/FeOOH photoanode before PEC test*

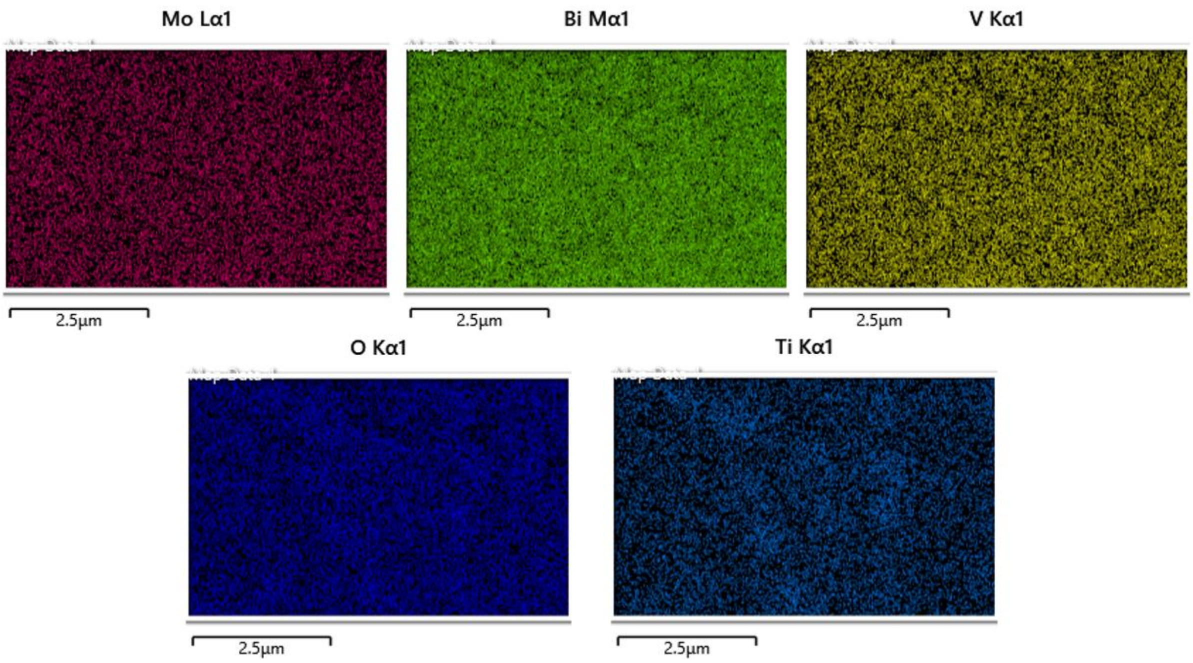

Figure S17, *EDS Colour mapping of Mo-$BiVO_4$/TiO_2/FeOOH photoanode after PEC test*

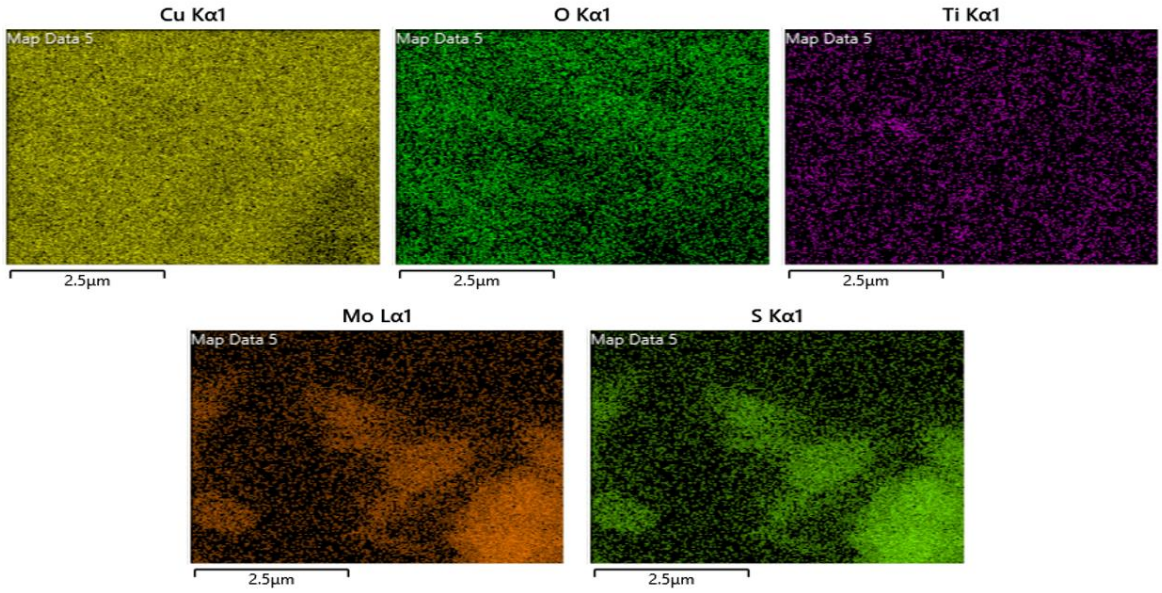

Figure S18, *EDS Colour mapping of Cu_2O/TiO_2/MoS_2 photocathode before PEC test*

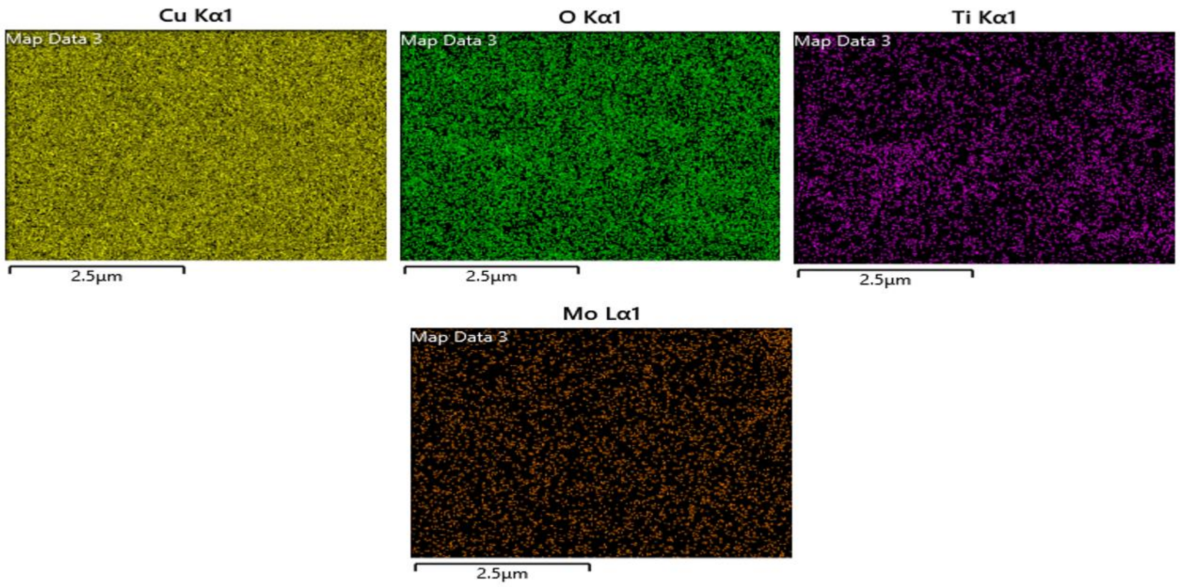

Figure S19, *EDS Colour mapping of $Cu_2O/TiO_2/MoS_2$ photocathode after PEC test*

4.3 X-ray photoelectron spectroscopy (XPS) analysis

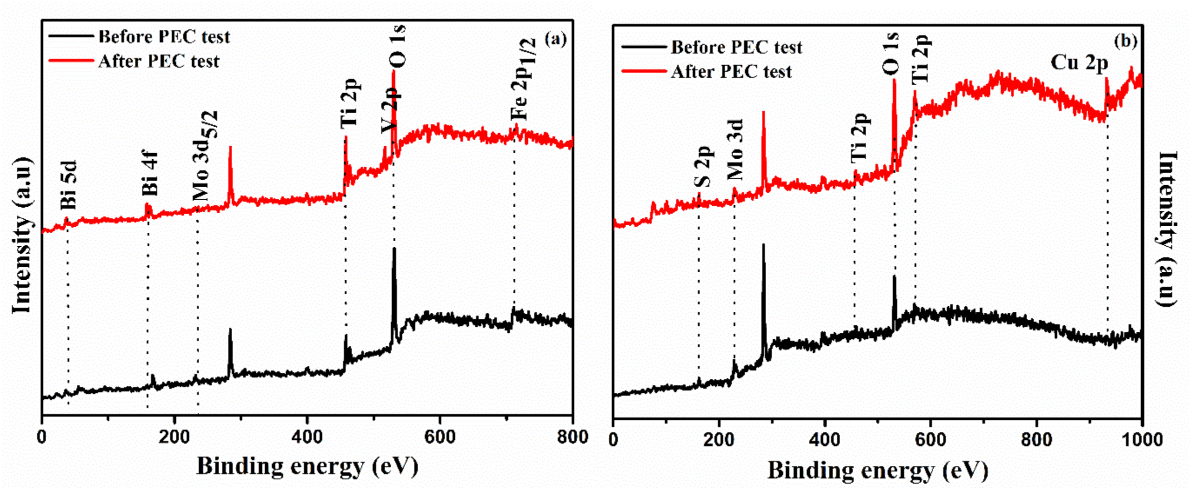

Figure S20, *XPS survey spectra of (a) Mo-$BiVO_4/TiO_2$/FeOOH photoanode and $Cu_2O/TiO_2/MoS_2$ photocathode before and after PEC test*

The XPS spectra was analyzed for Mo-$BiVO_4/TiO_2$/FeOOH photoanode and $Cu_2O/TiO_2/MoS_2$ photocathode before and after PEC test to understand the chemical states. The survey spectrum indicates the presence of Mo, Bi, V, O, Ti and Fe in figure S20 (a), which originates from Mo-$BiVO_4/TiO_2$/FeOOH photoanode. Figure S21 shows the core level XPS spectra of Mo 3d, Bi 4f, V 2p, O1s, Ti 2p and Fe 2p. The Mo 3d peaks at 232.38 eV and 236.63 eV before and after PEC tests was assigned to Mo $3d_{5/2}$ and Mo$3d_{3/2}$, respectively confirming Mo^{6+} oxidation state [34]. The Bi 4f spectrum presents two peaks before and after PEC test around binding energies of 158.99 eV and 164.32 eV corresponding to Bi $4f_{7/2}$ and Bi $4f_{5/2}$, respectively and the result proves the presence of Bi^{3+} oxidation state. The V 2p spectrum peaks around 516.90 eV and 524.37 eV before and after PEC test was assigned to V $2p_{3/2}$ and V $2p_{1/2}$ which represents V^{5+} oxidation state[35]. The O 1s spectrum exhibits two peaks at 530.34 eV and 531.80 eV which are

doublet peaks because of lattice O and surface hydroxyl group (Fe – OH) on $BiVO_4$ [36]. It was observed that surface hydroxyl group peak at 531.80 eV is reduced in intensity after PEC test due to loss of FeOOH catalyst. The observed binding energies signify the core level formation of $BiVO_4$. The Ti 2p spectrum yields peaks at 458.87 eV and 464.45 eV corresponding to core level of TiO_2 layer (Ti^{4+} oxidation state). The result proves the presence of TiO_2 layer on photoanode before and after PEC test. Binding energies peaks at 711.35 eV and 724.34 eV was attributed to Fe $2p_{1/2}$ and Fe $2p_{3/2}$ confirming Fe^{3+} oxidation state. The intensity of photoanode after PEC test is reduced which exhibits the lower content of FeOOH on the surface of photoanode.

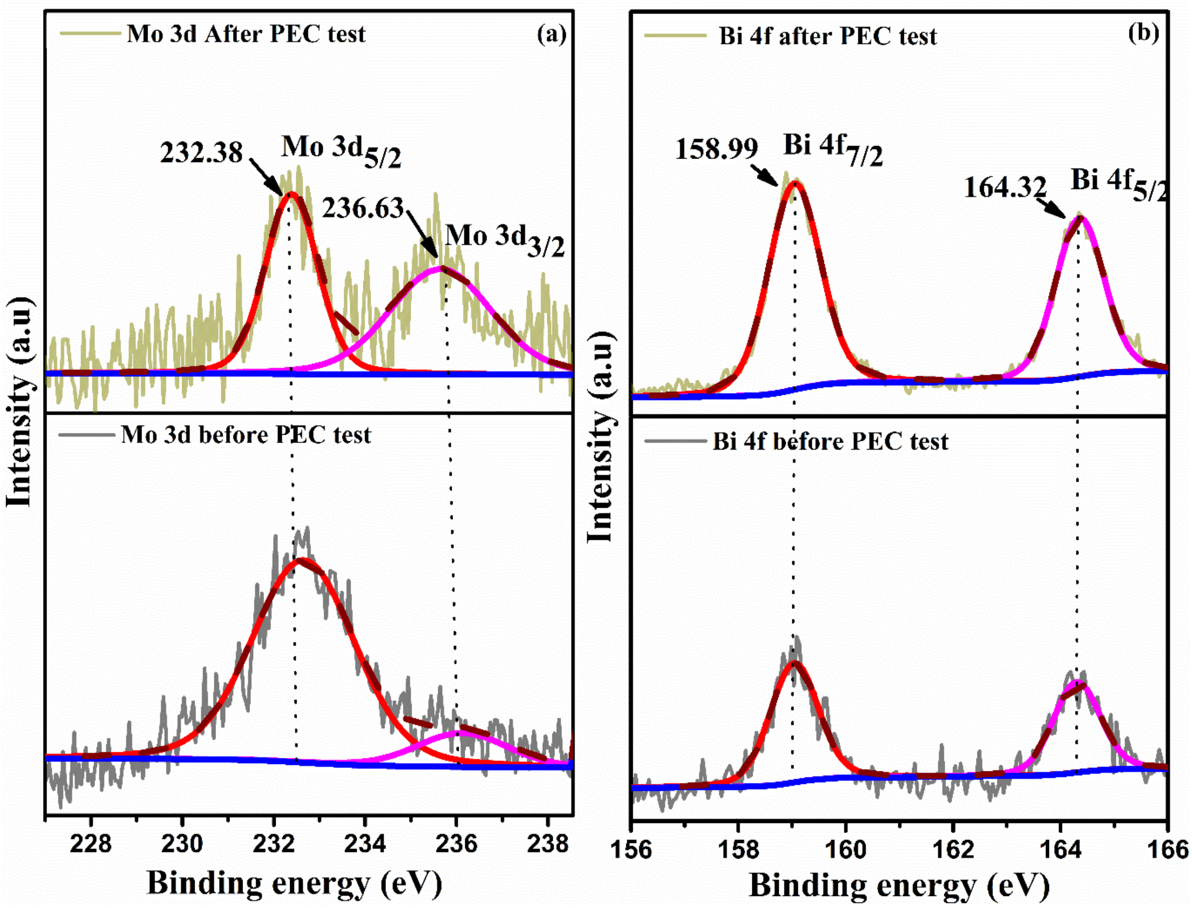

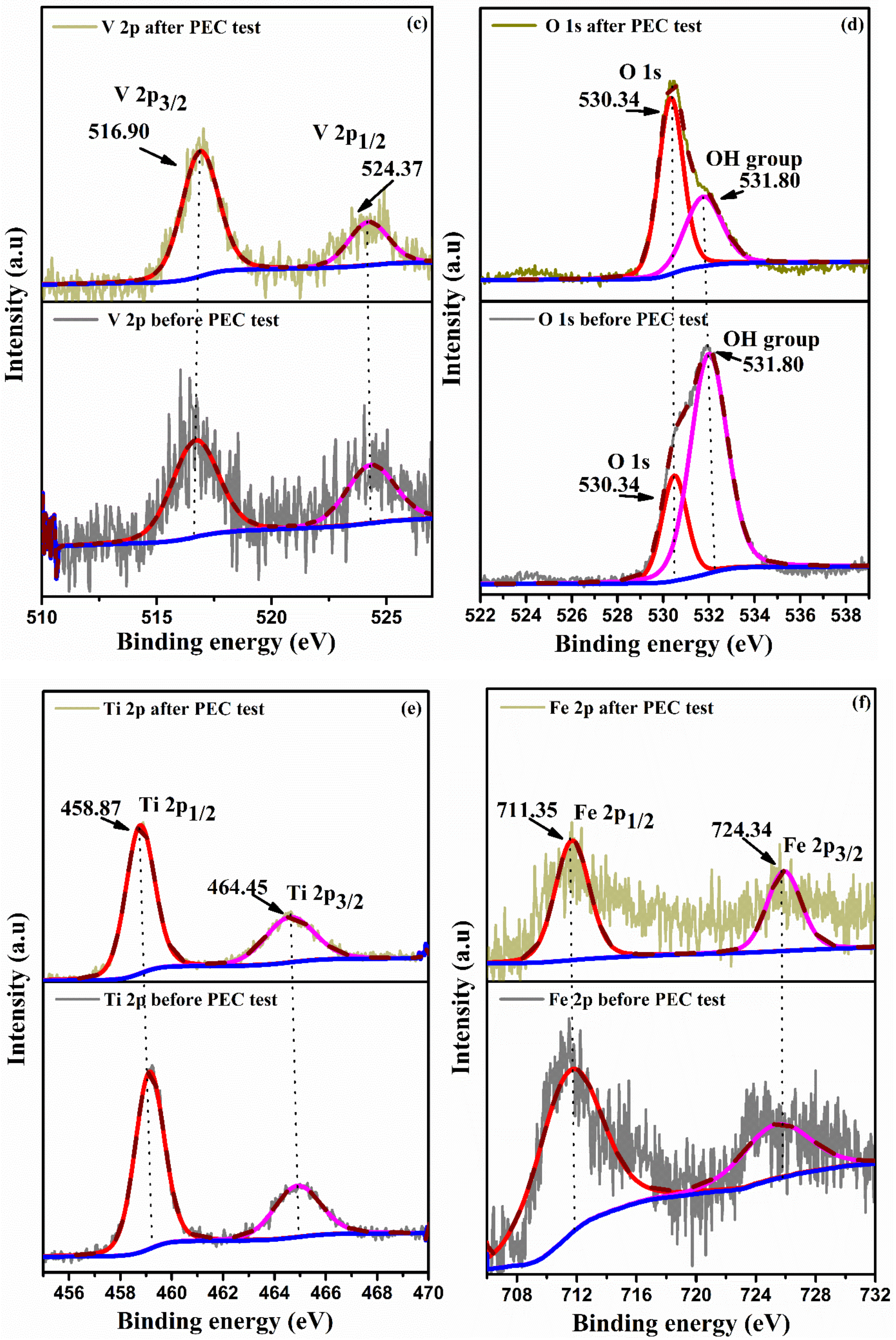

V 2p after PEC test
(c)
V 2p3/2
516.90
V 2p1/2
524.37
V 2p before PEC test
Intensity (a.u)
510
515
520
525
Binding energy (eV)
O 1s after PEC test
(d)
O 1s
530.34
OH group
531.80
O 1s before PEC test
OH group
531.80
O 1s
530.34
Intensity (a.u)
522
524
526
528
530
532
534
536
538
Binding energy (eV)
Ti 2p after PEC test
(e)
458.87
Ti 2p1/2
464.45
Ti 2p3/2
Ti 2p before PEC test
Intensity (a.u)
456
458
460
462
464
466
468
470
Binding energy (eV)
Fe 2p after PEC test
(f)
711.35
Fe 2p1/2
724.34
Fe 2p3/2
Fe 2p before PEC test
Intensity (a.u)
708
712
716
720
724
728
732
Binding energy (eV)

Figure S21, *High resolution XPS spectra of Mo-BiVO4/TiO2/FeOOH photoanode performed before and after PEC test in which (a) Mo 3d, (b) Bi 4f, (c) V 2p, (d)O 1s, (e) Ti 2p and (f) Fe 2p of photoanode*

The survey spectrum in figure 20 (b) indicates the presence of Cu, O, Ti, Mo and S for $Cu_2O/TiO_2/MoS_2$ photocathode. Figure S22 (a to d) shows the core level XPS spectra of Cu 2p, O 1s, Ti 2p, Mo 3d of Cu_2O, TiO_2 protective layer and MoS_2 catalyst before and after the PEC test. Before PEC test, the observed Cu 2p spectra at 932.96 eV and 952.75 eV are ascribed to Cu $2p_{3/2}$ and Cu $2p_{1/2}$. The result signifies the presence of Cu^+ oxidation state in Cu_2O [37]. After PEC test, in addition to the existing the Cu^+ oxidation state, the Cu^{2+} oxidation state of CuO is also observed at 935.05 eV in the XPS spectra due to possibly photooxidation of Cu_2O [38,39]. The result indicates that the TiO_2 protection layer might have some non-uniformity as well as porosity. The O 1s spectrum exhibits a single peak at 530.95 eV which is the lattice oxygen of Cu_2O[40]. The presence of TiO_2 layer before and after PEC test was confirmed by the peaks at 458.29 eV and 464.32 eV which corresponds to Ti $2p_{1/2}$ and Ti 2p state from core level TiO_2. The oxidation state was Ti^{4+} for Ti in TiO_2 layer. The presence of MoS_2 was confirmed by Mo 3d spectrum with peak at 232.28 eV and 235.71 eV which points 2H phase of MoS_2 [41]. The intensity of Mo $3d_{3/2}$ peaks are greatly reduced after PEC test which signifies the loss of MoS_2 catalyst after PEC test. The presence of peaks at 161.47 eV and 162.78 eV confirms the presence of sulfur in MoS_2 before and after the PEC test [42].

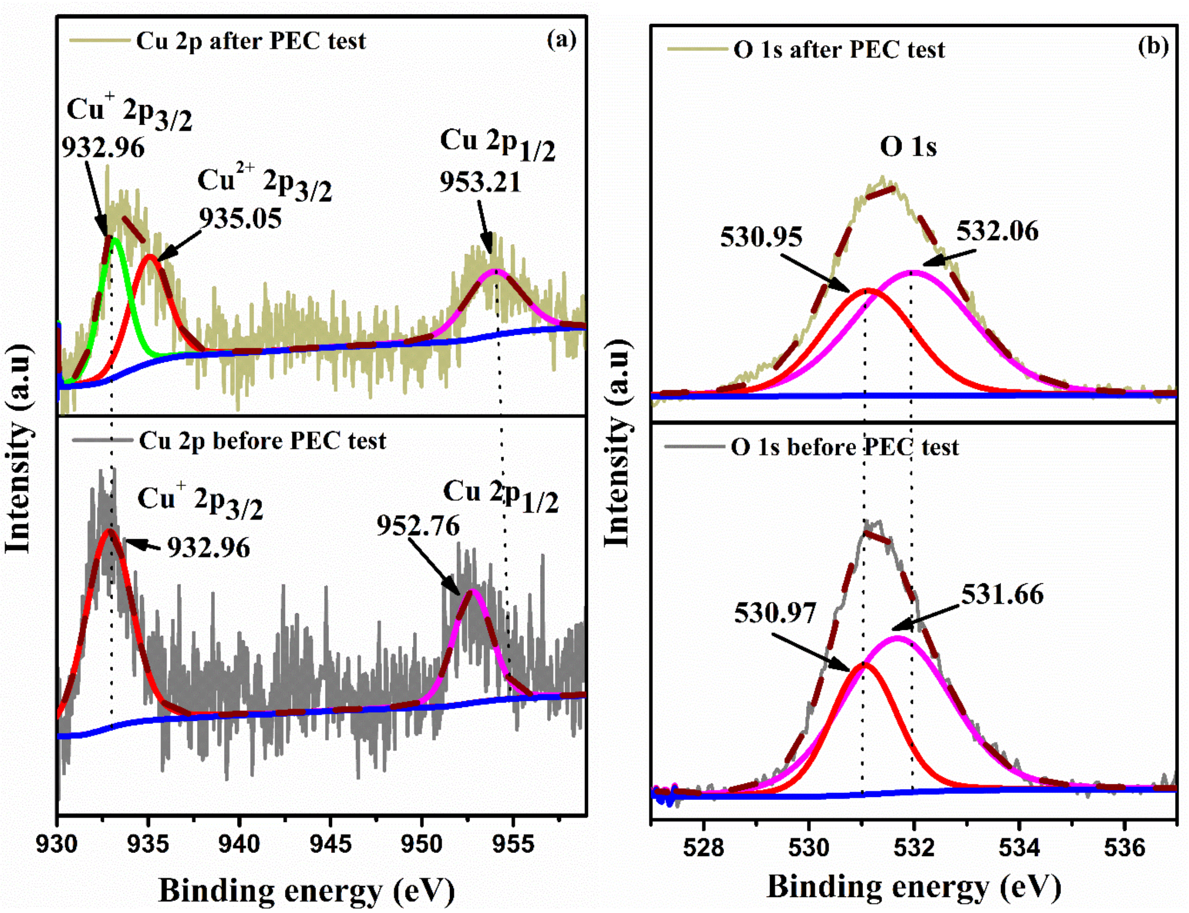

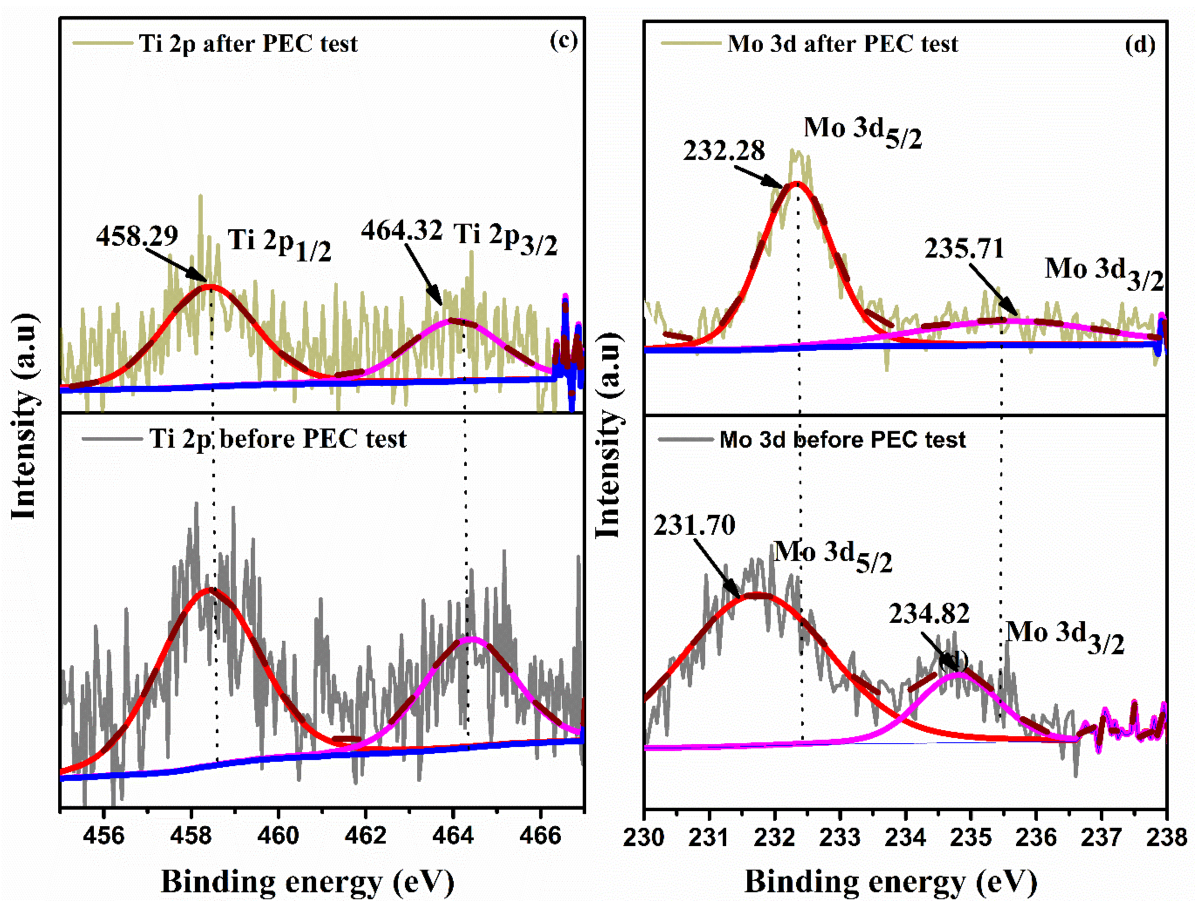
Ti 2p after PEC test
(c)
458.29
Ti 2p1/2
464.32
Ti 2p3/2
Ti 2p before PEC test
Intensity (a.u)
456
458
460
462
464
466
Binding energy (eV)
Mo 3d after PEC test
(d)
Mo 3d5/2
232.28
235.71
Mo 3d3/2
Mo 3d before PEC test
231.70
Mo 3d5/2
234.82
Mo 3d3/2
Intensity (a.u)
230
231
232
233
234
235
236
237
238
Binding energy (eV)

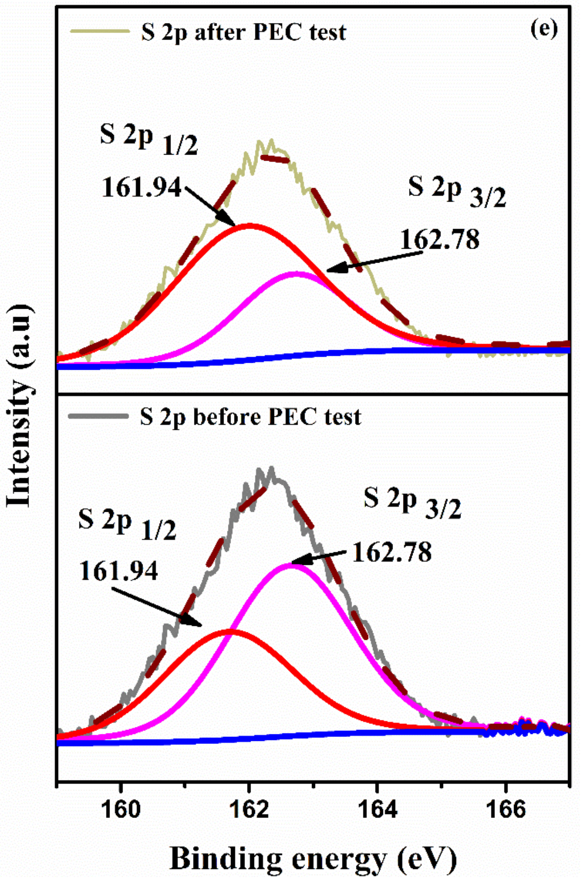
S 2p after PEC test
(e)
S 2p 1/2
161.94
S 2p 3/2
162.78
S 2p before PEC test
S 2p 3/2
S 2p 1/2
162.78
161.94
Intensity (a.u)
160
162
164
166
Binding energy (eV)

Figure S22, *High resolution XPS spectra of $Cu_2O/TiO_2/MoS_2$ photocathode performed before and after PEC test in which (a) Cu 2p, (b) O 1s, (c) Ti 2p and (d) Mo 3d and (e) S 2p of photocathode*

Reference:

1 X. Yin, Q. Liu, Y. Yang, Y. Liu, K. Wang, Y. Li, D. Li, X. Qiu, W. Li and J. Li, *Int. J. Hydrogen Energy*, 2019, **44**, 594–604.

2 P. Bornoz, F. F. Abdi, S. D. Tilley, B. Dam, R. Van De Krol, M. Graetzel and K. Sivula, *J. Phys. Chem. C*, 2014, **118**, 16959–16966.

3 S. Bai, J. Han, K. Zhang, Y. Zhao, R. Luo, D. Li and A. Chen, *Int. J. Hydrogen Energy*, 2022, **47**, 4375–4385.

4 M. S. Sudi, L. Zhao, Q. Wang, Y. Dou, X. Shen, A. Wang and W. Zhu, *Appl. Surf. Sci.*, 2022, **606**, 154753.

5 S. S. Kalanur, Y. J. Lee and H. Seo, *Appl. Surf. Sci.*, 2021, **562**, 150078.

6 C. Zhou, Z. Sanders-Bellis, T. J. Smart, W. Zhang, L. Zhang, Y. Ping and M. Liu, *Chem. Mater.*, 2020, **32**, 6401–6409.

7 Y. Taga, Z. Pan, K. Katayama and W. Y. Sohn, *ACS Appl. Energy Mater.*, 2022, 3–8.

8 E. Park, S. S. Patil, H. Lee, V. S. Kumbhar and K. Lee, *Nanoscale*, 2021, **13**, 16932–16941.

9 W. Han, H. Lin, F. Fang, Y. Zhang, K. Zhang, X. Yu and K. Chang, *Catal. Sci. Technol.*, 2021, **11**, 7598–7607.

10 E. J. Joo, G. Park, J. S. Gwak, J. H. Seo, K. Y. Jang, K. H. Oh and K. M. Nam, *J. Nanomater.*, , DOI:10.1155/2016/1827151.

11 X. Liang, J. Xie, J. Xiong, L. Gong and C. M. Li, *Sustain. Energy Fuels*, 2018, **2**, 2053–2059.

12 L. Pan, J. H. Kim, M. T. Mayer, M. K. Son, A. Ummadisingu, J. S. Lee, A. Hagfeldt, J. Luo and M. Grätzel, *Nat. Catal.*, 2018, **1**, 412–420.

13 I. R. Hamdani and A. N. Bhaskarwar, *Sol. Energy Mater. Sol. Cells*, 2022, **240**, 111719.

14 T. Wang, Y. Wei, X. Chang, C. Li, A. Li, S. Liu, J. Zhang and J. Gong, *Appl. Catal. B Environ.*, 2018, **226**, 31–37.

15 Y. G. Lin, Y. K. Hsu, Y. C. Lin, Y. H. Chang, S. Y. Chen and Y. C. Chen, *J. Colloid Interface Sci.*, 2016, **471**, 76–80.

16 Y. K. Hsu, C. H. Yu, Y. C. Chen and Y. G. Lin, *Electrochim. Acta*, 2013, **105**, 62–68.

17 S. Tiwari, S. Kumar and A. K. Ganguli, *J. Photochem. Photobiol. A Chem.*, 2022, **424**, 113622.

18 D. Jeong, W. Jo, J. Jeong, T. Kim, S. Han, M. K. Son and H. Jung, *RSC Adv.*, 2022, **12**, 2632–2640.

19 N. Kaneza, P. S. Shinde, Y. Ma and S. Pan, *RSC Adv.*, 2019, **9**, 13576–13585.

20 C. Yang, P. D. Tran, P. P. Boix, P. S. Bassi, N. Yantara, L. H. Wong and J. Barber, *Nanoscale*, 2014, **6**, 6506–6510.

21 H. Qi, J. Wolfe, D. Fichou and Z. Chen, *Sci. Rep.*, , DOI:10.1038/srep30882.

22 T. Lai, C. Tsao, M. Fang, J. Wu, Y. Chang, Y. Chiu, P. Hsieh, M. Kuo, K. Chang and Y. Hsu, , DOI:10.1021/acsami.2c07145.

23 S. N. S. Nasir, N. A. Mohamed, M. A. Tukimon, M. F. M. Noh, N. A. Arzaee and M. A. M. Teridi, *Phys. B Condens. Matter*, 2021, **604**, 412719.

24 A. Song, P. Bogdanoff, A. Esau, I. Y. Ahmet, I. Levine, T. Dittrich, T. Unold, R. Van De Krol and S. P. Berglund, *ACS Appl. Mater. Interfaces*, 2020, **12**, 13959–13970.

25 X. Fu, H. Chang, Z. Shang, P. Liu, J. Liu and H. Luo, *Chem. Eng. J.*, , DOI:10.1016/j.cej.2019.122001.

26 Y. H. Lai, K. C. Lin, C. Y. Yen and B. J. Jiang, *Faraday Discuss.*, 2019, **215**, 297–312.

27 J. H. Kim, A. Adishev, J. Kim, Y. S. Kim, S. Cho and J. S. Lee, *ACS Appl. Energy Mater.*, 2018, **1**, 6694–6699.

28 M. Chen, Y. Liu, C. Li, A. Li, X. Chang, W. Liu, Y. Sun, T. Wang and J. Gong, *Energy Environ. Sci.*, 2018, **11**, 2025–2034.

29 P. Xu, J. Feng, T. Fang, X. Zhao, Z. Li and Z. Zou, *RSC Adv.*, 2016, **6**, 9905–9910.

30 D. Huang, K. Wang, L. Li, K. Feng, N. An, S. Ikeda, Y. Kuang, Y. Ng and F. Jiang, *Energy Environ. Sci.*, 2021, **14**, 1480–1489.

31 F. F. Abdi, L. Han, A. H. M. Smets, M. Zeman, B. Dam and R. Van De Krol, *Nat. Commun.*, 2013, **4**, 1–7.

32 H. Luo, C. Liu, Y. Xu, C. Zhang, W. Wang and Z. Chen, *Int. J. Hydrogen Energy*, 2019, **44**, 30160–30170.

33 A. Aktar, S. Ahmmed, J. Hossain and A. B. M. Ismail, *ACS Omega*, 2020, **5**, 25125–25134.

34 G. Talasila, S. Sachdev, U. Srivastva, D. Saxena and S. S. V Ramakumar, *Energy Reports*, 2020, **6**, 1963–1972.

35 D. A. Reddy, Y. Kim, H. S. Shim, K. A. J. Reddy, M. Gopannagari, D. Praveen Kumar, J. K. Song and T. K. Kim, *ACS Appl. Energy Mater.*, 2020, **3**, 4474–4483.

36 W. Zhang, J. Ma, L. Xiong, H. Y. Jiang and J. Tang, *ACS Appl. Energy Mater.*, 2020, **3**, 5927–5936.

37 M. Zhang, J. Wang, H. Xue, J. Zhang, S. Peng, X. Han, Y. Deng and W. Hu, *Angew. Chemie*, 2020, **132**, 18621–18625.

38 C. Y. Toe, Z. Zheng, H. Wu, J. Scott, R. Amal and Y. H. Ng, *Angew. Chemie - Int. Ed.*, 2018, **57**, 13613–13617.

39 P. Wang, Z. Liu, C. Han, X. Ma, Z. Tong and B. Tan, *J. Nanoparticle Res.*, , DOI:10.1007/s11051-021-05383-2.

40 A. A. Dubale, C. Pan, A. G. Tamirat, H. Chen, W. Su, C. Chen, J. Rick, D. W. Ayele, B. A. Aragaw, J. Lee, Y. Yang and B. Hwang, *J. Mater. Chem. A Mater. energy Sustain.*, 2015, **3**, 12482–12499.

41 G. Gupta, S. Palchoudhury, P. K. Kahol, S. R. Mishra and F. Perez, 2017, 1–12.

42 Q. Lin, X. Dong, Y. Wang, N. Zheng, Y. Zhao, W. Xu and T. Ding, *J. Mater. Sci.*, 2020, **55**, 6637–6647.
